# Lactylation-driven *NSUN2*-mediated RNA m5C modification promotes perineural invasion in pancreatic cancer

**DOI:** 10.7150/thno.122294

**Published:** 2026-01-01

**Authors:** Tianhao Huang, Chonghui Hu, Huimou Chen, Honghui Jiang, Tingting Li, Qing Tian, Rihua He, Yuan Yuan, Yong Jiang, Yu Zhou, Qing Lin, Zhihua Li, Mingming Xiao, Xuebiao Wei, Rufu Chen, Shangyou Zheng

**Affiliations:** 1 Guangdong Cardiovascular Institute, Guangdong Provincial People's Hospital, Guangdong Academy of Medical Sciences, Guangzhou, Guangdong, 510080, China.; 2 Department of Pancreas Center, Department of General Surgery, Guangdong Provincial People's Hospital (Guangdong Academy of Medical Sciences), Southern Medical University, Guangzhou, Guangdong, 510080, China.; 3 Guangdong Provincial People's Hospital (Guangdong Academy of Medical Sciences), Southern Medical University, Guangzhou, Guangdong ,510080, China.; 4 Department of Oncology, Sun Yat-sen Memorial Hospital of Sun Yat-sen University, Guangzhou, Guangdong, 510120, China.; 5 School of medicine, South China University of Technology, Guangzhou, Guangdong, 510006, China.; 6 Department of Geriatric Intensive Medicine, Guangdong Provincial Geriatrics Institute, Guangdong Provincial People's Hospital (Guangdong Academy of Medical Sciences), Southern Medical University, China.; 7 Department of Pancreatic Surgery, Fudan University Shanghai Cancer Center, Shanghai 200032, China.

**Keywords:** pancreatic ductal adenocarcinoma (PDAC), *NSUN2*, m5C, lactylation modification, perineural invasion

## Abstract

**Background:** Perineural invasion (PNI) is a key biological feature underpinning the high malignancy and poor prognosis of pancreatic ductal adenocarcinoma (PDAC). Lysine lactylation (Kla), a metabolite-stress-induced post-translational modification, plays crucial regulatory roles in diverse biological processes. The RNA methyltransferase *NSUN2* is essential for cancer invasion and metastasis. However, the mechanisms by which *NSUN2* contributes to lactylation-driven PNI in PDAC remain to be elucidated.

**Methods:** We assessed tumor lactate / pan-lactylation, *NSUN2* lactylation, and PNI in human PDAC cohorts with survival follow-up. Functional studies used PDAC cell lines for migration/invasion assays, dorsal-root-ganglion (DRG) co-culture, and neurite-outgrowth assays under lactate or enzymatic perturbations. Mechanistic interrogation combined *NSUN2* knockout, CRISPR knock-in mutants at K692 (K692R/E), co-immunoprecipitation, RIP-seq, MeRIP-qPCR, and actinomycin-D chase to test mRNA binding, m5C modification, and stability of *CDCP1*/*STC1*. *In vivo* validation employed a sciatic nerve invasion model and a KPC genetically engineered mouse model to assess tumor-nerve infiltration and disease progression.

**Results:** Lactylated *NSUN2* is markedly upregulated in mice and human PDAC with more severe PNI, and is significantly associated with poorer prognosis. Functionally, inhibiting lactylation or blocking *NSUN2* markedly attenuated tumor-nerve interactions and neural invasion. Mechanistically, lactate accumulation leads to the lactylation of *NSUN2* at lysine 692 (K692), subsequently inhibiting its ubiquitination and degradation. lactylation of *NSUN2* mediated m5C modification on *CDCP1* and *STC1* mRNA, enhanced their mRNA stability.

**Conclusions:** This study identifies lactate-driven *NSUN2* K692 lactylation as a key driver of perineural invasion in PDAC. We define a lactate-*NSUN2*-m5C-*CDCP1*/*STC1* axis that links metabolic stress-induced lysine lactylation to mRNA methylation-dependent stabilization of pro-invasive transcripts, highlighting actionable therapeutic targets to restrain neural invasion and improve patient outcomes.

## Introduction

Perineural invasion (PNI) represents a hallmark biological feature of pancreatic carcinoma, characterized by tumor cell tracking along perineural spaces of surrounding nerves, thereby promoting local dissemination and distant metastasis 1. Clinically, PNI correlates strongly with early tumor progression, postoperative relapse, and serves as an independent predictor of unfavorable prognosis. Pancreatic ductal adenocarcinoma (PDAC) exhibits an exceptionally high prevalence of PNI, observed in approximately 80-100% of cases 2. Early alterations in nerve architecture can already be observed at the preinvasive epithelial stage 3,4. Our earlier randomized controlled trial (RCT) revealed that integrating modified retroperitoneal nerve dissection into standard Whipple surgery markedly enhanced disease-free survival (DFS) among patients with pancreatic head cancer. Moreover, this surgical approach effectively mitigated postoperative back pain, underscoring the pivotal role of PNI as a therapeutic target in pancreatic cancer management 5.

RNA methylation, a form of epigenetic modification that preserves the gene sequence, is critical for regulating a wide range of biological functions. Proteins involved in this process are classified into three main groups: 'writers' that add methyl groups to RNA, 'erasers' that remove them, and 'readers' that identify and bind to these methylated RNA molecules. Although significant progress has been made in understanding the structure and pharmacology of RNA methylation regulators, the role of post-translational modifications (PTMs) in regulating these proteins has been largely overlooked. 5-Methylcytosine (m5C), a key epigenetic mark, is an RNA modification extensively found in eukaryotic ribosomal, messenger, transfer, and long non-coding RNAs. It has a vital role in regulating cellular functions by influencing RNA stability, nuclear export, translation efficiency, and processing. Accumulating evidence has revealed a complex link between m5C modification and cancer development 6-9, and our recent findings further demonstrate that cancer-associated fibroblasts (CAFs) can promote PNI in pancreatic cancer through extracellular vesicle (EV) delivery of lncRNA-*PIAT*, which mediates m5C modification 10. However, the precise role of m5C modifications in lactate metabolism-driven PNI in pancreatic cancer remains to be fully elucidated.

Lactate, a byproduct of glycolysis, has been shown to contribute to tumor progression by supplying energy during tumorigenesis and facilitates immune evasion by creating an acidic microenvironment 11-13. Beyond its established metabolic role, lactate has been identified as a key factor in generating a novel post-translational modification, lysine lactylation (Kla), which occurs on both histone and non-histone proteins 14-16. In PDAC, Li *et al.* revealed lactate-dependent histone lactylation, particularly H3K18la, plays a key role in driving pancreatic ductal adenocarcinoma progression through a glycolysis-linked positive feedback loop involving mitotic checkpoint regulators, suggesting novel targets for PDAC therapy 17. However, the effect of the novel Kla modification on the epigenetic landscape and its contribution to PNI in pancreatic cancer remains to be determined.

Our study focused on uncovering how lactate mediates neural infiltration in PDAC. Using both *in vivo* and *in vitro* systems, we observed that elevated lactate and lactylation levels markedly enhance the invasion of tumor cells towards nerves. Mechanistically, our results indicate that increased lactate induces lactylation of *NSUN2* at lysine 692, thereby inhibiting its ubiquitination and subsequent degradation. This stabilization of *NSUN2* leads to the upregulation of *CDCP1* and *STC1* mRNA via m5C-dependent mechanisms, ultimately driving PNI. These findings underscore the essential role of intracellular lactate in facilitating PNI through *NSUN2* lactylation and RNA m5C modification, providing a potential therapeutic strategy to target lactylation or m5C modification in the treatment of PDAC.

## Materials and Methods

### Patients and specimens

From December 2014 to October 2023, a total of 142 pancreatic ductal adenocarcinoma (PDAC) specimens were obtained from patients undergoing surgical resection at Guangdong Provincial People's Hospital. Eligible participants were those with histologically confirmed PDAC who had not received any preoperative radiotherapy or chemotherapy. Individuals with a history of previous malignancies or prior cancer-related treatments were excluded from enrollment. Patients lacking complete follow-up data were also omitted from analysis. The study population included participants of both sexes, and gender balance had no influence on the inclusion of clinical samples. Each participant signed informed consent documents, experimental protocols were reviewed and approved by the Guangdong Provincial People's Hospital's Ethics Committees.

PNI is defined by the occurrence of cancer cells along nerve (cells encircle roughly one-third or more of the neural perimeter, and / or penetrate the epineurium, perineurium, or endoneurium). The severity of PNI was determined based on both degree and frequency of PNI. The scoring for PNI degree was as: 0 (none), 1 (perineural, indicated that cancer cell encircle roughly one-third or more of the neural perimeter) and 2 (intraneural, indicated that cancer presented within epineural, perineurial, and endoneurial space of the neuronal sheath). The scoring for PNI frequency was as: 0 (none), 1 (low), 2 (frequent) and 3 (excessive). The overall PNI score was calculated as the product of its extent and occurrence frequency. The severity of PNI was evaluated by final score with a cut-off point of < 4 versus ≥ 4. Patients with severe PNI (score of 4-6) were divided into 'with PNI group' (PNI+) and others (score of 0-3) were divided into 'without PNI group' (PNI-).

### Multiplexed immunofluorescence

To perform multiplexed immunofluorescence, tissue sections were stained by PANO 4-plex IHC Kit (Panovue, China) following supplier's protocol. In brief, paraffin-embedded tissue slides were deparaffinized and rehydrated, followed by heat-induced antigen retrieval with EDTA buffer (pH 9.0). To minimize nonspecific reactions, endogenous peroxidase activity was quenched using a blocking reagent (Golden Bridge Biotechnology, China) for 10 min, after which the sections were further blocked with goat serum (Golden Bridge Biotechnology, China) for 15 min. Subsequently, the slides were treated with primary antibodies against *Tuj1* (D65A4, CST), *CK19* (D4G2, CST), Pan-lac (PTM-1401RM, PTM Biolabs), *NSUN2* (20854-1-AP, proteintech), *STC1* (20621-1-AP, proteintech) and *CDCP1* (12754-1-AP, proteintech)) for a 1 h incubation. After three washes with TBST, HRP - conjugated secondary antibodies were applied for 30 min, followed by fluorescent tyramide amplification for 15 min. Finally, nuclei were stained with DAPI, and fluorescence images were acquired with a Zeiss LSM900 confocal microscope (Carl Zeiss Microscopy).

### Immunofluorescence staining

For cell, PANC-1 cells cultured on confocal dishes were first treated with 4% paraformaldehyde for fixation at room temperature and then exposed to 0.5% Triton X-100 for permeabilization. After fixation and permeabilization, blocking was performed at 37 °C for 1 h in PBS supplemented with 1% BSA and 0.1% Triton X-100 to reduce background staining. The primary antibodies, prepared in the same blocking buffer, were added to the cells and incubated overnight at 4 °C. After three washes with PBS, dye-conjugated secondary antibodies diluted in blocking buffer were applied for 1 h away from light. Following another three PBS washes, the nuclei were counterstained with DAPI for 10 min. Fluorescence images were acquired with a Zeiss LSM900 confocal microscope (Carl Zeiss Microscopy, Germany).

### Western blot analysis

Cell samples are lysed on ice for 20 min in RIPA buffer supplemented with protease and phosphatase inhibitors at a dilution of 1:1:100. The lysates were then centrifuged at 12,000 rpm for 30 min at 4 °C, and the resulting supernatant were collected. Protein concentration were quantified using a BCA Protein assay kit. Equal amounts of protein samples were separated by SDS-PAGE according to molecular weight and electrotransferred onto polyvinylidene fluoride (PVDF) membranes (Millipore, USA). The membranes were blocked with a quick blocking solution (epizyme, China) for 30 min at room temperature and subsequently incubated overnight at 4 °C with the corresponding primary antibodies. After washing with TBST buffer three times, HRP - conjugated secondary antibodies were applied 1 h at room temperature. Protein bands were detected using a Chemi XT4 imaging system. The list of antibodies used in this study is provided in Table S1.

### Lactate concentration measurement

After centrifugation at 12,000 × g for 10 min, the supernatant of the cell culture was collected. Lactate production was measured using a Lactate Assay Kit (Solarbio, Beijing, China) according to the manufacturer's instructions. The absorbance was recorded at 570 nm using a spectrophotometer, and lactate levels were normalized to cell number.

### Neural invasion *in vitro* model

To evaluate cell migration and invasion, pretreated PANC-1 cells (2 × 10^5^) were plated in 200 µL of DMEM without serum containing or lacking 30 mM L-lactate (Sigma-Aldrich, Cat#L6402). The lower chambers contained DMEM with 10% FBS. After 10 h, non-migrated cells were carefully wiped off, and migrated cells were fixed (4% paraformaldehyde, 10 min) and stained (crystal violet, 10 min). Cell numbers were determined microscopically to evaluate cell migratory and invasive activity.

For DRG cells' neurite length analysis, pretreated tumor cells (2 × 10^5^) were placed in the upper compartment of a 6-well Transwell apparatus containing a 0.4-µm filter membrane, and primary DRG cells (2 × 10^5^) were placed in lower chamber. The total nerve axon length per square millimeter was recorded by phase-contrast microscopy at 6, 12, 18, 24, 30, 36, 42, and 48 h.

For the co-culture analysis of DRG and tumor cells, Panc-1 cells were resuspended in Matrigel (Invitrogen, USA) and seeded in 10 μL onto a 6-well plate. DRG explants isolated from neonatal rats were placed 3 mm away from the Panc-1 cells in 10 μL of Matrigel. After allowing the Matrigel to solidify by incubating at 37 °C for 20 min, the designated conditioned medium containing or lacking 30 mM L-lactate (Cat#L6402, Sigma-Aldrich), which was replenished every 48 h. Phase-contrast microscopy was acquired on first and seventh day of coculture with an optical imaging system (Invitrogen, USA). The neural invasion index was determined according to the following formula: 1 - (distance between the tumor front and DRG on seventh day) / (distance between the tumor front and DRG on first day).

### Dorsal root ganglia (DRGs) and DRG cells isolation from neonatal Sprague-Dawley rats

After humane euthanasia of neonatal Sprague-Dawley rats, the spinal cords were quickly dissected, and DRGs were carefully collected and placed into F12 medium (Gibco, USA) for subsequent use. To obtain single DRG cells, the isolated ganglia were enzymatically dissociate at 37 °C for 30 min in a digestion buffer collagenase (Sigma-Aldrich, USA) and trypsin (Sigma-Aldrich, USA). After digestion, the resulting cell suspension was washed with DMEM supplemented with 10% fetal bovine serum and centrifuged. The collected cell pellet was resuspended in fresh medium for further analysis.

### m5C dot blot assay

After treating wild-type or *NSUN2*-knockout PANC-1 cells with ddH2O or L-lactate for 2 days, total RNA was extracted and diluted uniformly to 300 ng/μL. The samples were denatured at 95 °C for 3 min and immediately cooled on ice. Then, 2 μL of the sample was spotted onto an NC Transfer Membrane (Merck Millipore, Germany) and cross-linked under UV light for 20 min. After washing the membrane with TBST buffer, it was blocked in buffer for 1 h at ambient temperature. Primary antibody (anti-m5C, 1:5000; Proteintech) was applied overnight at 4 °C, followed by secondary antibody incubation for 1 h at ambient temperature. After 5 min ECL development, chemiluminescent bands were captured using a Chemi XT4 detection unit.

### qRT-PCR assay

Total RNA was isolated from cultured cells and tissue specimens using the EZB-RN4 extraction kit (EZB Bioscience). Complementary DNA (cDNA) was synthesized from purified RNA with the PrimeScript RT Reagent Kit (Vazyme, China) according to the manufacturer's protocol. Quantitative real-time PCR was then performed using the Vazyme SYBR-based system. Relative mRNA expression levels were calculated with the 2^-ΔΔCT^ algorithm. Primer sequences used in this study are listed in Table S1.

### Immunoprecipitation assay

Cell samples are lysed on ice for 15 min using IP lysis buffer containing protease and phosphatase inhibitors, prepared at a dilution ratio of 1:1:100. The lysate is centrifuged at 13,000 g for 10 min at 4 °C. After centrifugation, the supernatant is incubated overnight at 4 °C with the target antibody or IgG antibody. Protein A/G magnetic beads (Invitrogen, USA), pre-washed with wash buffer, are added to the mixture and incubated at room temperature for 1 h. After washing the beads with wash buffer, 100 μL of elution buffer (Invitrogen, USA) is added, and the mixture is left at room temperature for 10 min. The precipitated proteins are then analyzed by Western blotting.

### Mass spectrometry

Protein samples obtained from co-immunoprecipitation (CO-IP) were resolved by SDS-PAGE and visualized using Coomassie Brilliant Blue staining. The protein bands showing differential intensity were excised and submitted to APTBIO (Shanghai, China) for mass spectrometric analysis. Lyophilized peptide samples were redissolved in double-distilled water containing 0.1% formic acid, and 2 μL of each solution was injected onto a nanoViper C18 trap column (Acclaim PepMap 100, 75 μm × 2 cm). Peptide separation was performed on a nanoElute system (Bruker) with Solvent A (0.1% formic acid in 99.9% water) and Solvent B (0.1% formic acid in 99.9% acetonitrile). The samples were initially loaded in 95% solvent A and then separated on an EASY-nLC analytical column (Thermo Scientific, 15 cm × 150 μm, 3 μm, C18) using a 30 min gradient at a flow rate of 300 nL/min: 5% - 35% B for 18 min, 35% - 80% B for 2 min, and 80% B for 10 min. Mass spectrometric detection was carried out on a timsTOF Pro instrument (Bruker) operated in positive ion mode. The electrospray voltage was set to 1.5 kV, and precursor as well as fragments were analyzed using a TOF mass range of m/z 100 - 1700. The timsTOF Pro was run in parallel accumulation serial fragmentation (PASEF) mode, and data were acquired using the following parameters: 1 MS scan followed by 8 MS/MS PASEF scans per cycle; total cycle time = 0.95 s; ion charge range = 0 - 5; and active exclusion enabled with a release duration of 24 s.

### RNA immunoprecipitation sequencing (RIP-seq)

RIP was carried out using the EZ-Magna RIP kit (Millipore, USA). Briefly, after removing DNA from the cell lysate, immunoprecipitation is performed by incubating the lysate with the target antibody at 4 °C overnight for RNA-protein complex formation. Following this, equilibrated A/G magnetic beads are added, and the mixture is incubated at 4 °C for 1 h. Finally, RNA is extracted using the Trizol method. Finally, the precipitated RNAs were purified for deep sequencing. Purified RNA was subjected to 2 × 150 bp paired-end sequencing (PE150) on an illumina Novaseq™ 6000 platform (LC-Bio Technology CO., Ltd., Hangzhou, China) following standard procedures. Specifically, differential peak analysis between the experimental and control groups was performed using MAnorm. This software first merges peaks identified independently in the experimental and control IP samples, and then performs differential analysis based on the number of reads mapped to the merged peaks in each group. Differentially expressed genes (DEGs) were further identified with DESeq2, applying thresholds of Fold Change > 2 and p < 0.05.

### UV cross-linking and immunoprecipitation (CLIP)

The CLIP assay was performed using the EZ-Magna RIP kit (Millipore, USA) in accordance with the manufacturer's instructions. Initially, cells were pre-treated and subjected to UV crosslinking at 254 nm with a dose of 400 mJ/cm², followed by lysis using RIP lysis buffer. The resultant lysates were then sonicated, and RNase I (Life Technologies) along with Turbo DNase (Life Technologies) was applied. Subsequently, magnetic beads, pre-coated with 1 μg of the specified antibody or anti-IgG, were introduced to the cell lysate and incubated overnight at 4 °C. The RNA precipitated from this mixture was released through treatment with proteinase K and subsequently analyzed by qRT-PCR.

### m5C quantification (m5 C-RIP-qPCR)

The m5C levels in indicated transcripts were measured by m5C RNA immunoprecipitation followed by qRT-PCR. Briefly, total RNA is extracted from specified cells and purified into mRNA using the Dynabeads mRNA Purification Kit (Ambion, USA). The mRNA fragments are incubated overnight at 4 °C with an anti-m5C antibody (ab10805, Abcam) or control IgG antibody, followed by a 2 h incubation with Protein A/G magnetic beads at 4 °C. After three washes, RNA is purified using the Trizol method, then analyzed by qRT-PCR. Ten percent of the mRNA fragments are used as input, with primers listed in Table S1.

### RNA stability assay

For RNA stability analysis, the indicated cells were incubated with 5 μg/mL actinomycin D and harvested at the indicated time points (0, 3, 6, 9 and 12h). Total RNA was extracted and isolated and quantified by qRT-PCR. The mRNA half-life was determined by fitting the decay curve using a non-linear regression model (one phase decay) in GraphPad Prism.

### Animal experiments

To establish the sciatic nerve invasion model, PANC-1 cells were preincubated with 30 mM L-lactate for 48 hours and transfected with shRNA against *NSUN2* or a non-targeting control (shNC). BALB/c nude mice (4 weeks old, n = 15) were anesthetized and positioned prone to allow surgical access to the sciatic nerves. A total of 2 μL of tumor cell suspension (1 × 10⁴ cells/μL) was injected into the right sciatic nerve using a Hamilton syringe, and as a negative control, the left sciatic nerve received an injection of the same volume of PBS. Sciatic nerve function was monitored weekly, which was defined on a 1-4 scale: A score of 4 indicated normal hind-limb motor function with full toe and ankle extension and symmetric gait, 3 indicated mild impairment with slight weakness or reduced toe spreading, 2 represented moderate dysfunction with partial foot drop and reduced voluntary movement, and 1 denoted complete paralysis with loss of voluntary motion and no measurable muscle contraction upon stimulation. To quantitatively assess paralysis severity, two complementary metrics were employed: hind-limb muscle strength was measured using a grip-strength meter. Every two weeks, the hindpaw toe-spread (first-fifth digits) was recorded to assesse the sciatic nerve index of mice. After 6 weeks, all mice were euthanized, and sciatic nerve and tumor specimens were collected and fixed in 4% paraformaldehyde for histological analysis.

### Genetically engineered mouse model

Genetically engineered KPC mice (LSL-KRAS G12D/+; LSL-TP53 R172H/+; PDX-1-CRE+/+) were purchased from Shanghai Model Organisms Center. The mice were intraperitoneally injected with sodium lactate (1 g/kg, n = 15) once daily, and were injected with AAV packaging sh*NSUN2* (n = 15) . After a 4-week experimental period, the mice were euthanized, and pancreatic tissues were harvested, fixed in 4% paraformaldehyde, and embedded for histological analysis.

### Immunohistochemistry

Paraffin-embedded tissue sections were deparaffinized and rehydrated, followed by antigen retrieval in EDTA buffer for 21 min. The sections were then incubated at room temperature with a peroxide block for 10 min. Subsequently, the tissue sections were incubated overnight at 4 °C with the primary antibody against *NSUN2*, pan-lac, *CDCP1* or *STC1*. Afterward, signal enhancement solution and polymer-conjugated HRP-labeled goat anti-mouse/rabbit IgG were applied and incubated at 37 °C for 20 min. The antigen location was visualized using DAB substrate, and counterstaining with hematoxylin was performed for contrast.

### Plasmid construction, RNA interference, lentiviral transduction, and CRISPR-Cas9 editing

All plasmids and siRNAs used in this study were synthesized by IGE Biotechnology (Guangzhou, China), and lentiviral and CRISPR-Cas9 systems were provided by Genecefe Biotechnology (Jiangsu, China). For plasmid construction, full-length human *NSUN2* and its lysine-to-arginine variants (K692R, K441R, K640R, K712R, K257R) were inserted into the pcDNA3.1 expression vector. Full-length human *NSUN2* was also subcloned into a FLAG-tagged expression plasmid, while full-length Ubiquitin and GFP were ligated into an HA-tagged vectors. CRISPR-Cas9 technology was applied to establish stable *NSUN2* knockout cell lines, methylation-domain mutants, and lysine-substituted variants (K692R, K692E, K692A, and K692Q). Short hairpin RNAs (shRNAs) targeting *NSUN2* were designed in the pCDH-CMV-MCS-EF1-Puro backbone (sequences listed in Table S1). Plasmids transfections were carried out using Lipofectamine 3000 and P3000 reagents (Invitrogen, USA). For siRNA-mediated silencing, Lipofectamine 3000 was employed to knock down *LDHA*, *LDHB*, and *NSUN2* (see Table S1 for sequences). Lentiviral-mediated gene delivery was performed following the provider's protocol, and resistant cells were maintained under puromycin pressure (A3740, APExBIO, USA) for two weeks to generate stable populations.

### Generation of *NSUN2* point-mutant PANC-1 cell lines

CRISPR-Cas9 ribonucleoprotein (RNP)-mediated homology-directed repair (HDR) with single-stranded oligodeoxynucleotide (ssODN) donors was employed to introduce K692E, K692R, K692Q, and K692A substitutions at the endogenous *NSUN2* locus. The DepMap database was consulted to ensure that the selected cell line harbored an *NSUN2* copy-number state suitable for genome editing. Three sgRNAs targeting the vicinity of K692 were designed and computationally screened for on- and off-target efficiency. Cas9 protein and sgRNA were preassembled into RNP complexes, combined with the corresponding ssODN donor, and delivered into PANC-1 cells via electroporation. After 48-72 h, single-cell cloning was performed, and colonies were expanded in 96-well plates. Genomic DNA was extracted and PCR-amplified using primers flanking the target exon. Positive clones were initially screened by allele-specific PCR and/or restriction fragment length polymorphism (RFLP) analysis, followed by Sanger sequencing to confirm precise substitutions, zygosity, and silent mutations introduced for PAM disruption. Verified homozygous clones were expanded and subjected to quality control prior to downstream experiments. All sgRNA and ssODN sequences used in this study are listed in Table S1.

### Statistical analysis

All data were performed by SPSS software (version 27.0) and GraphPad Prism (version 9.0). Quantitative results were presented as mean ± standard deviation (SD) from at least three independent experiments unless stated otherwise. Differences between two groups were assessed using either Student's t-test or the non-parametric Mann-Whitney U test according to the data distribution. For comparisons among multiple groups, one-way or two-way analysis of variance (ANOVA) was used, followed by Tukey's multiple comparison test to evaluate intergroup differences. Non-parametric categorical variables were examined by Chi-square or Fisher's exact test. The correlation between continuous variables was evaluated using Spearman's rank correlation analysis. Cox model analysis provided likelihood-ratio-based p values, and Kaplan-Meier survival plots were created with the 'survival' package to display overall survival patterns. Associations among tissue lactate concentration, *NSUN2*, pan-lactylation, *CDCP1*, *STC1* expression, and clinical outcomes (overall survival, OS; disease-free survival, DFS) were analyzed by applying a multivariate Cox proportional-hazards model, and p values below 0.05 were interpreted as statistically significant.

### Ethics statement

All experimental procedures involving human tissues and animals were reviewed and approved by the Ethics Committee of Guangdong Provincial People's Hospital (approval number: KY2024-999-01). The study protocol was conducted in accordance with the principles of the Declaration of Helsinki and relevant national regulations. Written informed consent was waived as per approved protocol version (V1.0, dated May 1, 2024). Animal studies were conducted following institutional guidelines, and the animal experiment protocol (V1.0, dated July 17, 2023) was approved by the committee.

## Results

### Elevated lactate and lactylation levels are associated with poor prognosis and PNI in PDAC

**Figure 1:**
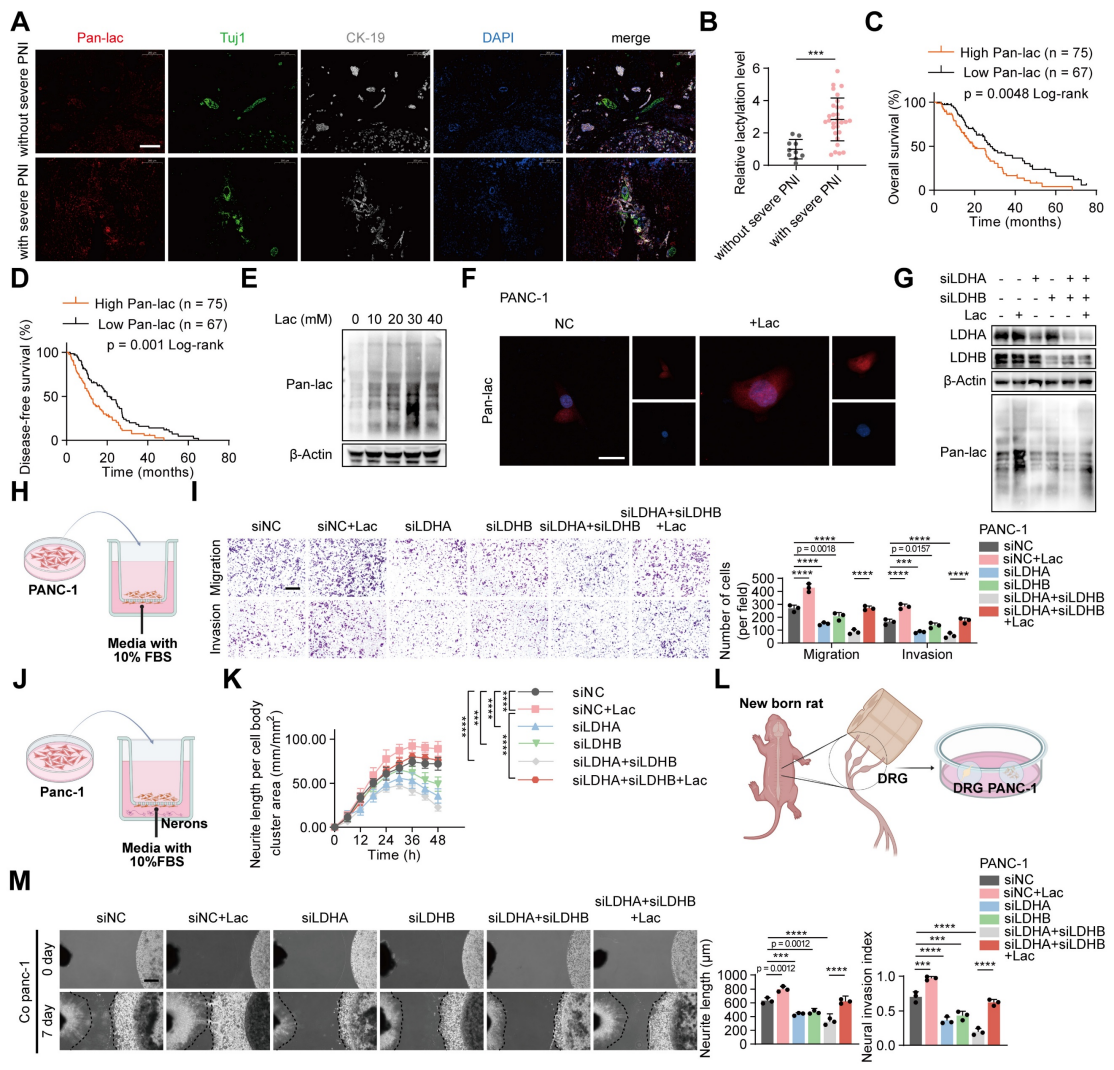
** Elevated lactate and lactylation levels are associated with poor prognosis and PNI in PDAC. (A)** Representative mIF staining for Pan-lac, *Tuj1* and *CK-19* expression in PDAC tissues grouped by PNI severity. Pan-lac reports lactylation; *Tuj1* labels neurons; *CK-19* identifies epithelial cancer cells. Scale bars, 200 μm. **(B)** Quantification of tissue lactylation levels in PDAC cases with or without severe PNI. **(C-D)** Kaplan-Meier survival curves showing OS **(C)** and DFS **(D)** in the PDAC cohort stratified by Pan-lac level (high vs low). **(E)** Immunoblot analysis of lactylation modification levels in PANC-1 cells following treatment with varying concentrations of L-lactate. **(F)** Immunofluorescence detection of Pan-lac in tumor cells treated as indicated. Scale bar, 20 μm. **(G)** PANC-1 cells subjected to *LDHA*/*LDHB* knockdown and L-lactate exposure were analyzed by Western blot to determine lactylation levels. **(H-M)** PANC-1 cells expressing siNC, *LDHA* siRNA, *LDHB* siRNA, or expressing both *LDHA* and *LDHB* siRNAs, followed by ± L-lactate treatment for subsequent experiments. **(H)** Schematic diagram of tumor cells in a Transwell assay. **(I)** PANC-1 Transwell migration/invasion: image panels (left) and measurements (right). Scale bar, 200 μm. (n = 3; one-way ANOVA; mean ± SD). **(J)** Schematic representation of the neuronal-tumor Transwell co-culture system. **(K)** Quantitative analysis of neurite outgrowth under the indicated conditions in the Transwell co-culture model. Phase-contrast images were obtained at 6 h intervals. (n = 3; two-way ANOVA; mean ± SD). **(L)** Schematic representation of the direct co-culture model between tumor cells and dorsal root ganglia (DRG). **(M)** (top) Representative fields from DRG co-cultures with tumor cells. (bottom) Summary statistics for tumor neurite invasion toward DRG. The black dashed line on the left indicates the growth boundary of the DRG, which on the right marks the growth boundary of PANC-1 cells. Scale bar, 500 μm. (n = 3; one-way ANOVA test; mean ± SD). ****P < 0.0001, ***P < 0.001, **P < 0.01, *P < 0.05.

To elucidate the role of lactylation in perineural invasion (PNI), we assessed global lactylation in tumors from patients with and without severe PNI. Immunofluorescence analysis demonstrated that patients with severe PNI exhibited markedly elevated levels of overall lactylation compared with those without (Figure 1A-B). Moreover, elevated global lactylation levels were associated with poorer overall survival (OS) and disease-free survival (DFS) (Figure 1C-D). Given that lactate serves as a substrate for lactylation, we next examined its effect on lactylation in PANC-1 cells. Western blot analysis showed that lactylation levels increased in a concentration-dependent manner, peaking at 30 mM L-lactate and declining at higher concentrations (Figure 1E, Figure S1A). Functionally, 30 mM L-lactate significantly enhanced the invasive and migratory abilities of PANC-1 cells and promoted neurite outgrowth in neuronal cells (Figure S1B-C). Consistently, Dorsal root ganglia (DRG) assays showed that 30 mM L-lactate markedly promoted tumor cell invasion into the DRG and facilitated neurite extension (Figure S1D). Lactate treatment also significantly increased intracellular lactate and global lactylation levels (Figure 1F, Figure S1E). Supporting these findings, primary tumor cells isolated from patients with severe PNI exhibited higher global lactylation than those from patients without PNI (Figure S1F), and displayed greater invasive and migratory properties as well as stronger neurite-promoting activity (Figure S1G-H). DRG assays further confirmed that primary tumor cells from patients with severe PNI showed enhanced invasion into DRG and induction of neurite outgrowth compared with cells from patients without severe PNI (Figure S1I).

To assess whether inhibiting lactylation could suppress neural invasion in pancreatic cancer, we decreased global lactylation in PANC-1 cells by transfecting siRNAs targeting *LDHA* and *LDHB*. Western blot analysis showed that knockdown of *LDHA* and *LDHB* effectively reduced global lactylation levels (Figure 1G, Figure S1J). Supplementation with L-lactate restored lactylation levels in *LDHA*/B-deficient PANC-1 cells (Figure 1G, Figure S1J). Functional assays demonstrated that *LDHA*/B knockdown significantly reduced the invasive and migratory abilities of PANC-1 cells, as well as neurite outgrowth in neuronal cells. Consistently, DRG assays revealed that *LDHA*/B knockdown markedly impaired the cells' ability to invade DRG and promote neurite outgrowth (Figure S2A-F). Notably, these effects were reversed by L-lactate supplementation (Figure 1H-M). To exclude the possibility that these phenotypic changes were attributable to altered cell proliferation, we compared colony formation and CCK-8 assays between control and lactate-treated cells under identical conditions, and found no significant differences (Figure S2G-H), indicating that the enhanced invasive behavior was not due to changes in cell growth. Collectively, these results indicated that lactylation play a critical role in PNI of pancreatic cancer.

### Lactate upregulates m5C and *NSUN2* levels to promote PNI in PDAC

**Figure 2:**
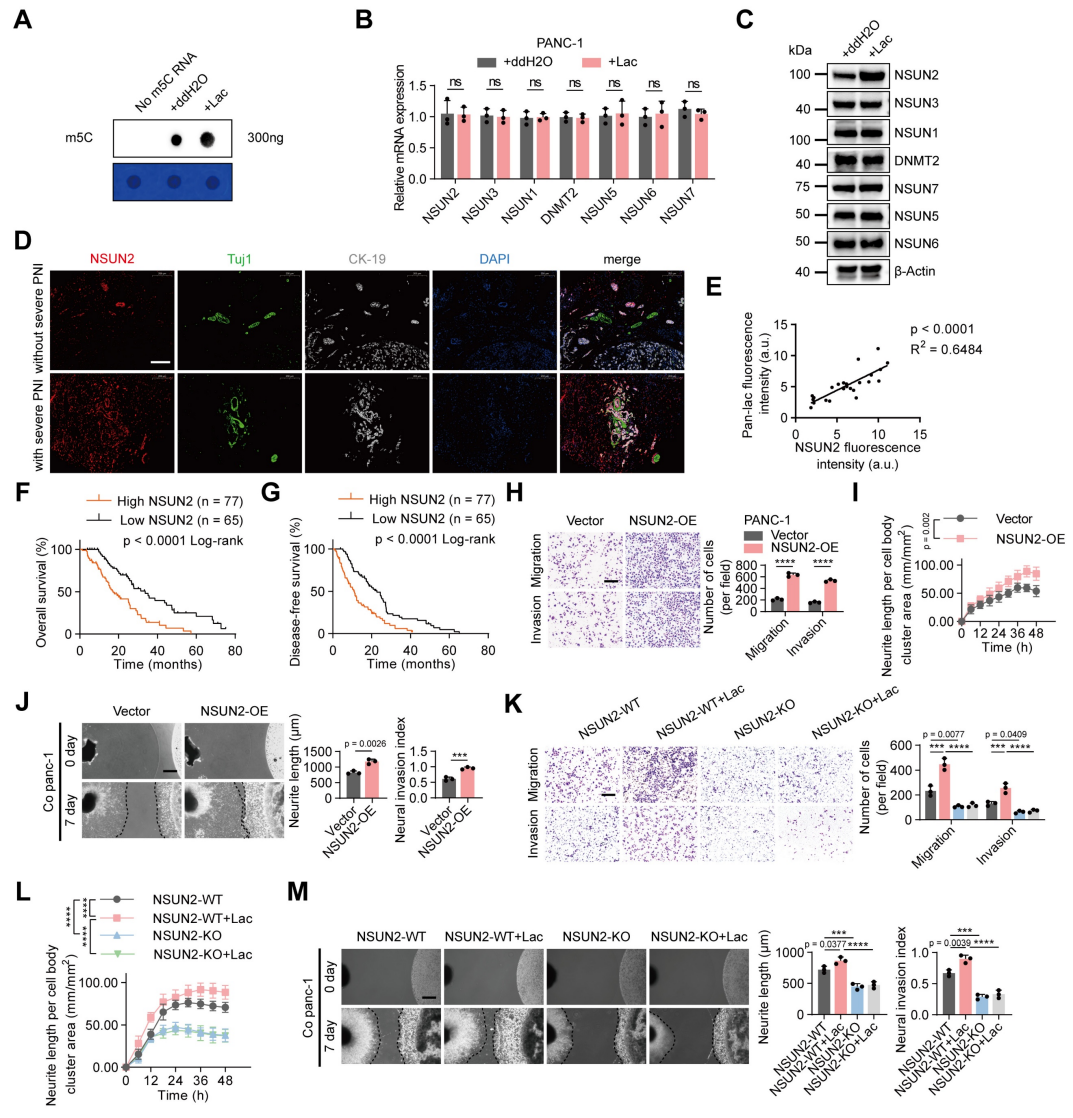
** Lactate upregulates m5C and *NSUN2* levels to promote PNI in PDAC. (A)** Dot blot analysis showing m5C modification levels in total RNA isolated from PANC-1 cells treated with or without L-lactate. Methylene blue staining (below) indicates RNA loading, whereas the dot blot signal intensity (above) reflects the global m5C content. **(B)** Quantitative RT-PCR analysis of *NSUN1*, *NSUN2*, *NSUN3*, *NSUN5*, *NSUN6*, *NSUN7* and *DNMT2* mRNA expression levels in PANC-1 cells followed by ± L-lactate treatment. **(C)** Immunoblot analysis of *NSUN1*, *NSUN2*, *NSUN3*, *NSUN5*, *NSUN6*, *NSUN7* and *DNMT2* protein expression levels in PANC-1 cells under the same treatment conditions. **(D)** Representative mIF staining for *NSUN2*, *Tuj1* and *CK-19* grouped by PNI severity in PDAC tissues. *Tuj1* marks neuronal cells, and *CK-19* identifies tumor epithelial cells. Scale bars, 200 μm. **(E)** Correlation between *NSUN2* expression levels and lactylation modification in 24 cases of PDAC tissues. Pearson correlation analysis was applied to estimate the coefficient (R) and significance level (P). **(F-G)** Kaplan-Meier survival curves showing OS **(F)** and DFS **(G)** in the PDAC cohort stratified by *NSUN2* level (high vs low). **(H-J)** Functional tests were performed on PANC-1 cells transduced with either control vector or *NSUN2*-OE plasmid. **(H)** Transwell-based migration/invasion: image panels (left) and measurements (right). Scale bar, 200 μm. (n = 3; unpaired t test; mean ± SD). **(I)** Neurite extension in the Transwell co-culture was quantified under the specified conditions; phase-contrast images were acquired at 6-h intervals. (n = 3; paired t test; mean ± SD). **(J)** (left) Representative fields from DRG co-cultures with tumor cells. (right) Summary statistics for tumor neurite invasion toward DRG. Scale bar, 500 μm. (n = 3; unpaired t test; mean ± SD). **(K-M)** *NSUN2*-knockout PANC-1 cells were generated using the CRISPR/Cas9 system. Wild-type or *NSUN2*-knockout PANC-1 cells grouped by L-lactate treatment (with / without). **(K)** Transwell-based migration/invasion: image panels (left) and measurements (right). Scale bar, 200 μm. (n = 3; one-way ANOVA test; mean ± SD). **(L)** Neurite extension in the Transwell co-culture was quantified under the specified conditions; phase-contrast images were acquired at 6-h intervals. (n = 3; two-way ANOVA test; mean ± SD). **(M)** (left) Representative fields from DRG co-cultures with tumor cells. (right) Summary statistics for tumor neurite invasion toward DRG. Scale bar, 500 μm. (n = 3; one-way ANOVA test; mean ± SD). ****P < 0.0001, ***P < 0.001, **P < 0.01, *P < 0.05.

m5C modification is implicated in a broad spectrum of biological functions 18. Our previous studies revealed that patients with severe PNI showed a marked increase in total m⁵C levels compared with those without PNI, thereby contributing to the progression of pancreatic cancer-associated PNI 10. However, the relationship between m5C modification and lactylation remained unclear. Intriguingly, we found that that L-lactate treatment markedly increased global m5C levels in PANC-1 cells (Figure 2A). Given that multiple RNA methyltransferases contribute to m5C modification, we assessed the expression of a panel of enzymes in response to lactate. At the mRNA level, none of these enzymes exhibited significant alterations following lactate treatment (Figure 2B). Notably, lactate treatment significantly upregulated *NSUN2* protein levels, whereas the expression of the other enzymes remained unaffected (Figure 2C, Figure S3A). Consistently, immunofluorescence staining of patient samples showed that *NSUN2* expression was markedly higher in tumors with severe PNI than in those without (Figure 2D). Furthermore, correlation analysis confirmed a positive association between lactylation levels and *NSUN2* expression (Figure 2E). Importantly, Kaplan-Meier survival analysis demonstrated that patients with high *NSUN2* expression had significantly poorer overall survival and disease-free survival compared with those with low *NSUN2* expression (Figure 2F-G).

To further assess the role of *NSUN2* in PNI, we manipulated its expression in PANC-1 cells. Transwell and neurite outgrowth assays showed that *NSUN2* overexpression enhanced PANC-1 cell invasion and migration, as well as neurite extension in neuronal cells. DRG assays further confirmed that *NSUN2* overexpression promoted PANC-1 cell invasion into DRG and facilitated neurite outgrowth (Figure 2H-J). Conversely, *NSUN2* depletion or treatment with an *NSUN2* small-molecule inhibitor suppressed PANC-1 cell invasion and migration, reduced neurite outgrowth in neuronal cells, and diminished the capacity of tumor cells to invade DRG and promote neurite extension. (Figure S3B-D). Notably, these effects were partially reversed rescued by L-lactate supplementation (Figure S3E-G). To further validate these findings, we generated an *NSUN2* knockout PANC-1 cell line. Under *NSUN2* knockout, the partial phenotypic rescue by L-lactate observed in *NSUN2*-depletion cells was largely abolished (Figure 2K-M). Importantly, re-expression of wild-type *NSUN2* in lactate-treated *NSUN2*-KO cells restored global m5C levels and re-established the perineural invasion phenotype (Figure S3H-K). By contrast, exogenous L-lactate increased global protein lactylation irrespective of *NSUN2* status, indicating that *NSUN2* lactylation represents a PNI-specific regulatory mechanism rather than a general effect on pan-lactylation (Figure S3L). These findings underscore the critical role of *NSUN2* in lactate-mediated PNI.

### Lactylation of *NSUN2* inhibits its ubiquitination and degradation

**Figure 3:**
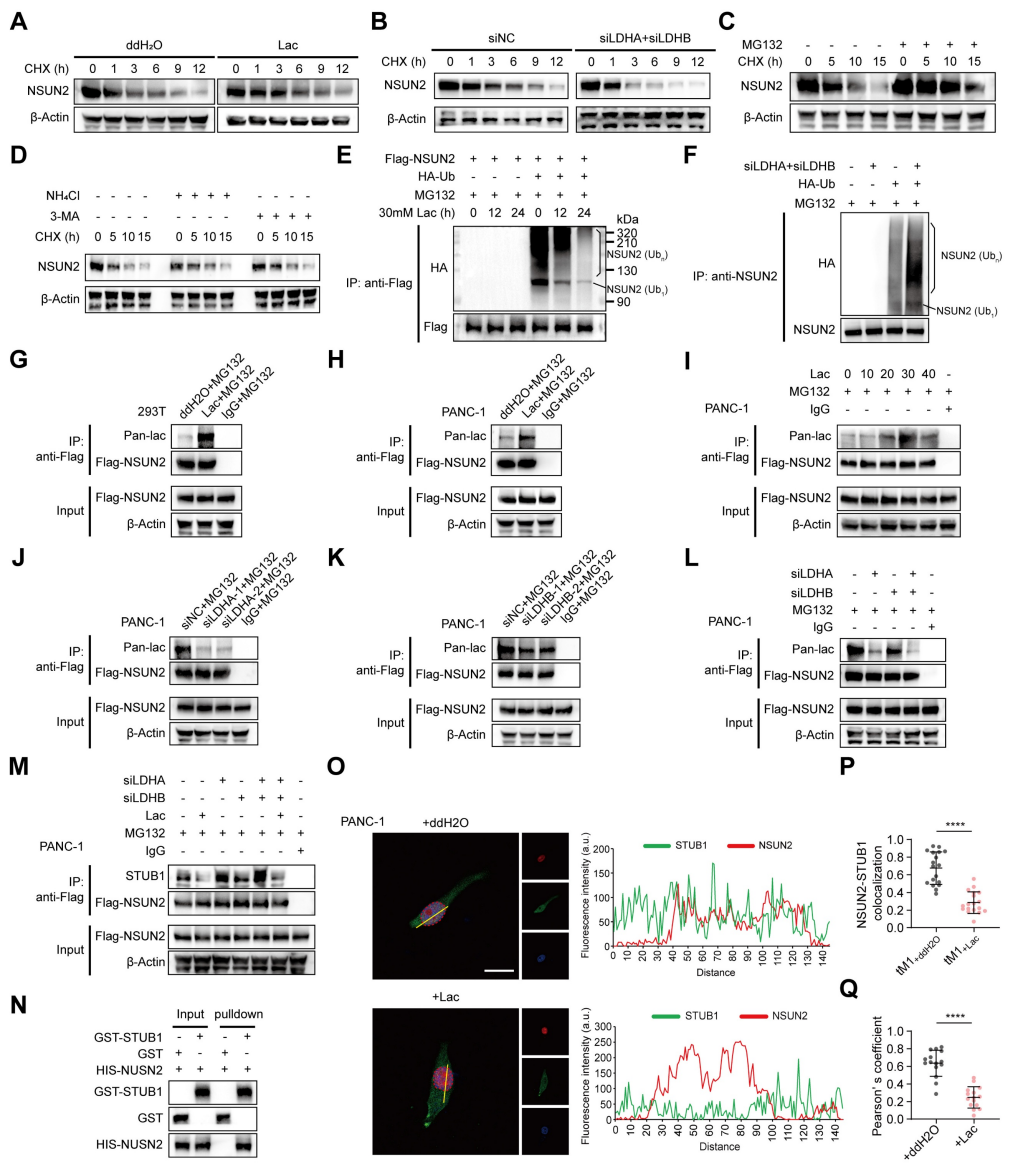
** Lactylation of *NSUN2* inhibits its ubiquitination and degradation. (A)** Immunoblot analysis of *NSUN2* in tumor cells exposed to ddH2O or L-lactate treatment, followed by treatment with cycloheximide (CHX) for specified time points.** (B)** Immunoblot analysis of *NSUN2* expression in PANC-1 cells carrying siNC or *LDHA*/*LDHB* siRNAs, followed by treatment with CHX for specified time points.** (C)** Immunoblot analysis of *NSUN2* in tumor cells exposed to MG132 treatment, followed by treatment with CHX for specified time points. **(D)** Immunoblot analysis of *NSUN2* in tumor cells exposed to NH_4_Cl or 3-MA treatment, followed by treatment with CHX for specified time points.** (E)** Following plasmid transfection, PANC-1 cells received MG132/L-lactate treatment for the specified intervals. Anti-FLAG immunoprecipitation of SDS-treated whole-cell lysates was performed, and *NSUN2* ubiquitination was evaluated by immunoblotting. The line labeled '*NSUN2* (Ub1)' indicates mono-ubiquitinated *NSUN2*, while the bracket labeled '*NSUN2* (Ubn)' marks high molecular weight polyubiquitinated *NSUN2* species. **(F)** PANC-1 cells expressing indicated plasmids and *LDHA*/*LDHB* siRNAs were treated with MG132. Anti-FLAG immunoprecipitation of SDS-treated whole-cell lysates was performed, and NSUN2 ubiquitination was evaluated by immunoblotting. **(G-H)** Lactylation of *NSUN2* in 293T cells **(G)** or PANC-1 cells **(H)** was confirmed using an immunoprecipitation (IP) assay. 293T cells or PANC-1 cells expressing FLAG-tagged *NSUN2* and were exposed to ddH2O or L-lactate treatment, with concurrent treatment with MG132. After lysis, SDS-treated extracts were subjected to anti-FLAG IP, followed by immunoblot detection. **(I)** Lactylation modification levels of *NSUN2* in PANC-1 cells following treatment with varying concentrations of L-lactate was confirmed using an immunoprecipitation (IP) assay. **(J-M)** Confirmation of *NSUN2* lactylation in PANC-1 cells expressing specific siRNAs by immunoprecipitation (IP). PANC-1 cells expressing FLAG-tagged *NSUN2* were treated with either ddH2O or L-lactate, followed by treatment with MG132. The cells were lysed for SDS pre-treated immunoprecipitation with anti-FLAG antibody followed by western blotting. **(J)** Lactylation of *NSUN2* in tumor cells expressing siNC or *LDHA* siRNA was confirmed through immunoprecipitation (IP) assay. **(K)** *NSUN2* lactylation in PANC-1 cells expressing siNC or *LDHB* siRNA was confirmed through immunoprecipitation (IP) assay. **(L)** *NSUN2* lactylation in PANC-1 cells expressing siNC, *LDHA* siRNA, *LDHB* siRNA, or both *LDHA* and *LDHB* siRNAs by immunoprecipitation (IP). **(M)** An immunoprecipitation blot showing the interaction levels between *NSUN2* and *STUB1* in PANC-1 cells transfected with siNC, *LDHA* siRNA, *LDHB* siRNA, or both *LDHA* and *LDHB* siRNAs and treated with ddH2O or L-lactate, using *NSUN2* antibody *in vitro*. **(N)** GST pull-down assay showing the interaction between *STUB1* and *NSUN2*. **(O)** (Left) Immunofluorescence detection of *NSUN2* and *STUB1* in tumor cells treated as indicated. (Right) Colocalization analysis of *NSUN2* and *STUB1* in the nuclei of PANC-1 cells under the same treatment conditions. The intensity profiles corresponding to the regions of interest (ROI, indicated by yellow lines) are shown. Red curve: *NSUN2* relative fluorescence intensity; green curve: *STUB1* relative fluorescence intensity. The overlap of the red and green curves indicates the colocalization of *NSUN2* and *STUB1*. Higher peak intensities correspond to greater levels of fluorescence expression. Scale bar, 20 μm. **(P-Q)** Manders' overlap coefficient (tM1) **(P)** and Pearson's correlation coefficient **(Q)** analysis of *NSUN2*-*STUB1* colocalization in tumor cells treated as indicated. (n = 15; unpaired t test; mean ± SD). ****P < 0.0001.

To test whether lactate regulates *NSUN2* stability, cycloheximide chase assays showed that exogenous L-lactate supplementation markedly prolonged the *NSUN2* half-life (Figure 3A, Figure S4A). Conversely, dual *LDHA*/*LDHB* knockdown, which reduces endogenous lactate, shortened the *NSUN2* half-life (Figure 3B, Figure S4B). Consistent with proteasome-mediated turnover, MG132 stabilized *NSUN2*, whereas lysosomal blockade with NH_4_Cl or 3-MA had no effect (Figure 3C-D, Figure S4C-E). We then interrogated ubiquitination directly. In cells co-expressing FLAG-*NSUN2* and HA-ubiquitin, exogenous L-lactate reduced the levels of polyubiquitinated *NSUN2* (Figure 3E). Conversely, *LDHA*/*LDHB* knockdown increased *NSUN2* ubiquitination (Figure 3F, Figure S4F). These results suggested that lactate regulates *NSUN2* stability through a post-translational mechanism, and indeed, we found that lactate induced robust lactylation of *NSUN2* (Figure 3G-I, Figure S4F-H). Moreover, this modification was markedly diminished following *LDHA*/B knockdown (Figure 3J-L, Figure S4I-K), confirming that both exogenous and endogenous lactate drive *NSUN2* lactylation. To delineate how lactate prevents *NSUN2* degradation, we focused on *STUB1* (STIP1 homology and U-box containing protein 1), an E3 ligase previously reported to mediate *NSUN2* degradation 19. Coimmunoprecipitation (Co-IP) assays revealed that lactate disrupted the interaction between *NSUN2* and *STUB1* (Figure 3M, Figure S4L). To exclude potential confounding from other cellular proteins, we performed GST pulldown assays with purified proteins, which confirmed that *NSUN2* directly binds to *STUB1* (Figure 3N). Immunofluorescence analysis further demonstrated that lactate disrupted the nuclear colocalization of *NSUN2* and *STUB1* (Figure 3O). Quantitative image analysis using Manders' colocalization coefficient (tM1) (Figure 3P) and Pearson's correlation coefficient (Figure 3Q) consistently showed that lactate treatment significantly disrupted *NSUN2*-*STUB1* colocalization. Collectively, these findings demonstrates that lactylation of *NSUN2* weakens the *NSUN2*-*STUB1* interaction, thereby preventing *STUB1*-mediated ubiquitination and degradation of *NSUN2*.

### *NSUN2* is lactylated at K692

**Figure 4:**
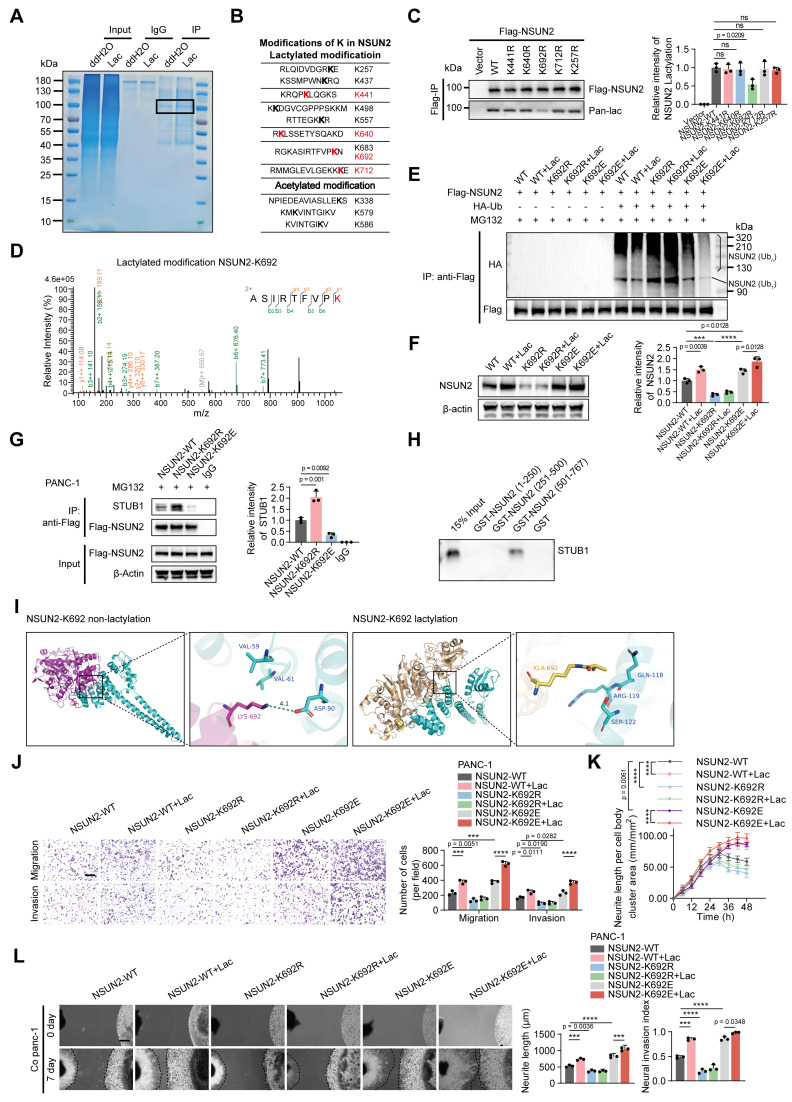
***NSUN2* is lactylated at K692. (A)** Representative Coomassie Brilliant Blue staining image of *NSUN2* protein from treated tumor cells. The boxed area marks NSUN2-specific bands analyzed by LC-MS. **(B)** Potential modifications of lysine (K) residues in *NSUN2* were assessed by mass spectrometry. Putative lactylation sites (upper panel) and acetylation sites (lower panel) are depicted. **(C)** (Left) PANC-1 cells were transfected with indicated *NSUN2* site mutations, followed by immunoprecipitation and and Western blotting to visualize NSUN2 lactylation with pan-lactyl and FLAG antibodies. (Right) Quantification of *NSUN2* lactylation intensity normalized to Flag-*NSUN2* (n = 3; mean ± SD; one-way ANOVA test). **(D)** Mass spectra for *NSUN2* peptides lactylated at K692. **(E-G)** *NSUN2*-K692 mutant PANC-1 cells were generated using the CRISPR/Cas9 approach, followed by treatment with or without L-lactate and were used for the subsequent experiments. Residue K692 was replaced by arginine (K692R) or glutamine (K692E). **(E)** Lysates from treated PANC-1 cells were SDS-pretreated, immunoprecipitated with anti-FLAG, and probed for *NSUN2* ubiquitination by Western blot. The line labeled '*NSUN2* (Ub1)' indicates mono-ubiquitinated *NSUN2*, while the bracket labeled '*NSUN2* (Ubn)' marks high molecular weight polyubiquitinated *NSUN2* species. **(F)** (Left) Whole-cell lysates made from PANC-1 cells under the indicated treatments were used for immunoblot to assess *NSUN2* protein expression. (Right) Quantification of *NSUN2* intensity normalized to *β-actin* (n = 3; mean ± SD; one-way ANOVA test). **(G)** (Left) Co-IP immunoblot illustrating the binding of *STUB1* to *NSUN2* in PANC-1 cells with various *NSUN2*-K692 mutations. (Right) Quantification of *STUB1* relative intensity (n = 3; mean ± SD; one-way ANOVA test). **(H)** *In vitro* binding of *STUB1* to various domains of *NSUN2*. The *in vitro* translated *STUB1* protein was incubated with GST, GST-*NSUN2* (1-250), GST-*NSUN2* (251-500), or GST-*NSUN2* (501-767) in GST pull-down assays. The glutathione eluates and 15% input materials were analyzed by SDS-PAGE followed by immunoblotting with anti-*STUB1* antibody. **(I)** Molecular docking analysis of *NSUN2*-*STUB1* interaction before and after *NSUN2* K692 lactylation. **(J-L)** *NSUN2*-K692 mutant PANC-1 cells were generated using the CRISPR/Cas9 approach, followed by treatment with or without L-lactate and were used for the subsequent experiments. **(J)** Transwell-based migration/invasion: image panels (left) and measurements (right). Scale bar, 200 μm. (n = 3; mean ± SD; one-way ANOVA test). **(K)** Quantitative evaluation of neurite outgrowth under the indicated conditions in the Transwell co-culture model. Phase contrast images were obtained at 6 h intervals. (n = 3; mean ± SD; two-way ANOVA test). **(L)** Schematic representation of the direct co-culture model between tumor cells and DRG. Scale bar, 500 μm. (n = 3; mean ± SD; one-way ANOVA test). ****P < 0.0001, ***P < 0.001, **P < 0.01, *P < 0.05.

To pinpoint the lactylation sites on *NSUN2*, we performed immunoprecipitation followed by LC-MS/MS, identifying nine lysine residues modified by lactylation. Among these, four sites (K441, K640, K692, K712) appeared only after lactate treatment and were absent in controls, suggesting that they are lactate-inducible modifications with potential functional relevance (Figure 4A-B). To determine their importance, we generated lysine-to-arginine substitutions at these four candidate sites, together with K257 as a nonresponsive control. Anti-Flag immunoprecipitation confirmed site-specific lactylation at K692 (Figure 4C). To further examine the functional relevance of this site, we compared K692 mutants with additional substitutions at K257, K441, K640, and K712. Migration, neurite outgrowth, and DRG co-culture assays revealed that mutations at K257, K441, K640, and K712 had minimal impact, whereas the K692R mutation markedly impaired these phenotypes (Figure S5A-C). This functional distinction was supported by LC-MS/MS analysis, which provided direct b-y ion evidence for lactylation at K692 (Figure 4D, Figure S5D-G). Substitution of K692 with non-lactylatable residues (K692R or K692A) enhanced *NSUN2* ubiquitination and reduced its protein abundance, whereas mimicking lactylation (K692E or K692Q) suppressed ubiquitination and stabilized *NSUN2* (Figure 4E-F, Figure S5H-I). We next examined how K692 lactylation influences the interaction between *NSUN2* and *STUB1*. Using CRISPR/Cas9-engineered PANC-1 cells carrying *NSUN2*-K692 mutants, co-immunoprecipitation assays showed that the K692E mutation, which mimics constitutive lactylation, markedly weakened the association of *NSUN2* with *STUB1*, whereas the non-lactylatable K692R mutant enhanced this interaction (Figure 4G). To further confirm the binding region between *NSUN2* and *STUB1*, GST pull-down assays were performed using recombinant *NSUN2* fragments (aa 1-250, 251-500, and 501-767). As expected, *STUB1* specifically bound to the C-terminal fragment GST-*NSUN2* (aa 501-767), but not to other regions or GST alone (Figure 4H). To further dissect the structural basis, molecular docking analyses revealed that K692 lactylation induces conformational changes that generate steric hindrance and alter electrostatic interactions at the *NSUN2*-*STUB1* interface, thereby impairing their binding (Figure 4I). Transwell and neurite outgrowth assays demonstrated that non-lactylatable mutants (K692R/A) significantly impaired tumor cell migration/invasion and reduced neurite outgrowth activity, whereas lactylation-mimetic mutants (K692E/Q) enhanced these phenotypes (Figure 4J-K; Figure S5J-K). Consistent with these findings, DRG assays corroborated that K692 lactylation promotes the perineural invasive behavior of pancreatic cancer cells (Figure 4L; Figure S5L). Together, these results identify K692 as the critical lactylation site on *NSUN2* that inhibits its ubiquitination, stabilizes the protein, and drives PNI in pancreatic cancer.

### *NSUN2* regulates *CDCP1* and *STC1* expression in an m5C-dependent manner

**Figure 5:**
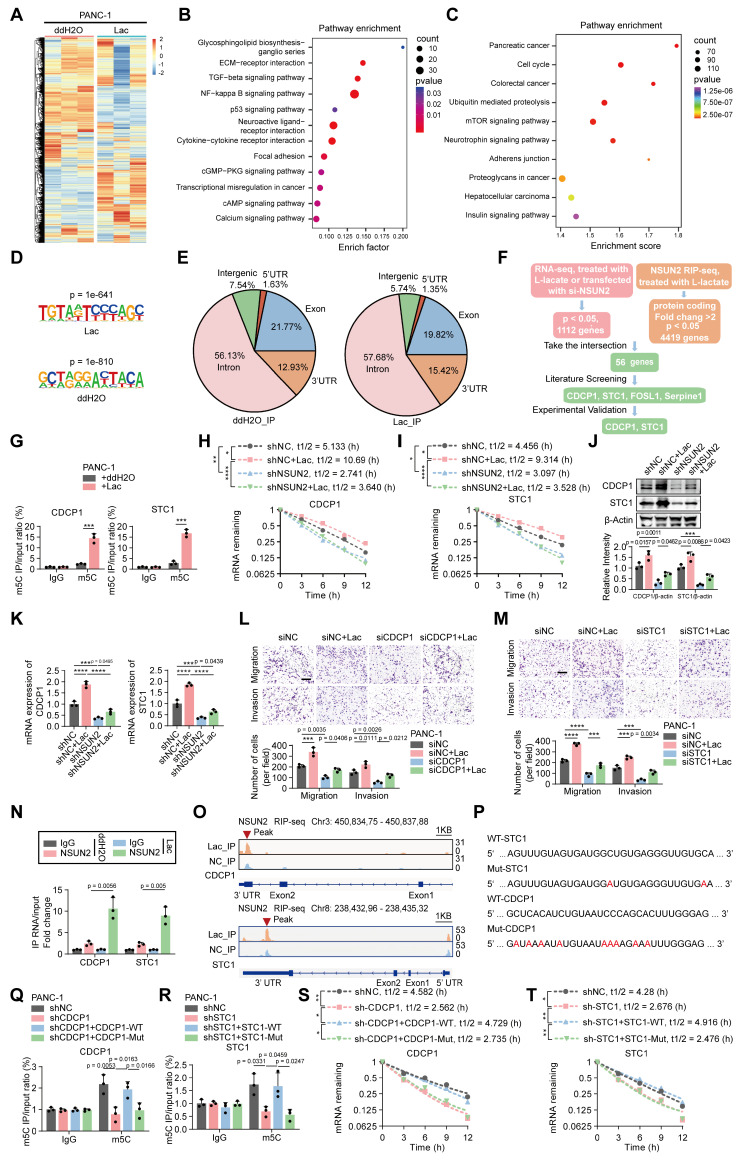
***NSUN2* regulates *CDCP1* and *STC1* expression in an m5C-dependent manner. (A)** Heatmap of mRNA changes in PANC-1 cells exposed to ddH2O or L-lactate treatment. **(B-C)** Pathway enrichment mapping (KEGG) in PANC-1 cells; dot size = pathway gene number, dot color = adjusted significance. **(B)** Pathway enrichment mapping (KEGG) of transcriptome sequencing between the control and lactate-treated groups. **(C)** Pathway enrichment mapping (KEGG) of *NSUN2* RIP-seq between the control and lactate-treated groups. **(D)** *NSUN2* binding Motif analysis of peak sequences using HOMER (v4.11.1) and visualization of nucleotide preferences. **(E)** *NSUN2* RIP-seq pie charts for both the ddH2O treatment (left) and L-lactate groups (right) depicting the distribution of *NSUN2* RIP-binding peaks. **(F)** Bioinformatic prediction pinpointed *CDCP1* and *STC1* as *NSUN2*-regulated genes. **(G)** MeRIP-qPCR confirmed m5C enrichment of *CDCP1* and *STC1* transcripts in PANC-1 cells ± L-lactate. **(H-I)** PANC-1 cells expressing shNC or *NSUN2*-specific shRNA, exposed to ddH2O or L-lactate treatment, were used for Actinomycin D assay to assess the half-life of *CDCP1* **(H)** and *STC1* **(I)** mRNA. **(J-K)** PANC-1 cells expressing shNC or *NSUN2*-specific shRNA, followed by specific treatment. **(J)** (Above) Immunoblot analysis of *CDCP1* and *STC1* in PANC-1 cell lysates prepared under the respective treatment conditions. (Below) Quantification of *CDCP1* and *STC1* intensity normalized to *β-actin* (n = 3; mean ± SD; one-way ANOVA test). **(K)** Total RNA isolated from treated PANC-1 cells was used for qRT-PCR analysis to assess the *CDCP1* and *STC1* mRNA expression. **(L-M)** PANC-1 cells expressing siNC, *CDCP1* siRNA **(L)** or *STC1* siRNA **(M)**, followed by treatment with or without L-lactate, were used for subsequent experiments. Transwell-based migration/invasion: image panels (top) and measurements (bottom). Scale bar, 200 μm. (n = 3; one-way ANOVA test; mean ± SD). **(N)** The interaction of *NSUN2* and the indicated mRNAs in PANC-1 cells exposed to ddH2O or L-lactate treatment was demonstrated by CLIP. **(O)** IGV tracks for *CDCP1* and *STC1* from RIP-seq analysis, peaks are marked with red triangles. **(P)** WT and potential m^5^C modification motif mutation *CDCP1*/*STC1* sequences were presented. Red fonts represented the mutant bases. **(Q-T)** WT-*CDCP1*, Mut-*CDCP1*, WT-*STC1* or Mut-*STC1* was overexpressed in *CDCP1*/*STC1* knockdown PANC-1 cells. **(Q-R)** MeRIP-qPCR analysis of *CDCP1* **(Q)** and *STC1* **(R)** mRNAs in PANC-1 cells under specific treatment conditions. **(S-T)** PANC-1 cells under specific treatment were used for Actinomycin D assay to assess the half-life of *CDCP1* **(S)** and *STC1* **(T)** mRNA. ****P < 0.0001, ***P < 0.001, **P < 0.01, *P < 0.05.

To identify the downstream targets of the *NSUN2* involved in PNI, we analyzed the mRNA expression profile of PANC-1 cells treated with L-lactate compared to control cells (Figure 5A). A total of 4419 differentially expressed genes (DEGs) were identified via mRNA sequencing (Figure 5A). Furthermore, KEGG pathway analysis indicated that increased lactylation levels upregulated signaling pathways associated with tumor invasion and metastasis (Figure 5B) 20,21. Considering that *NSUN2* functions as an m5C methyltransferase capable of selectively binding m5C-modified RNA, we next performed RNA immunoprecipitation sequencing (RIP-seq) in PANC-1 cells with or without lactate treatment. Pathway enrichment analysis of *NSUN2*-bound transcripts similarly highlighted signaling programs related to tumor invasion and neuronal interactions (Figure 5C). We mapped the binding motifs of *NSUN2*-associated mRNAs (Figure 5D) and confirmed that the *NSUN2* binding sites were predominantly enriched in the coding sequence (CDS) regions of mRNA transcripts, followed by the 3' UTR (Figure 5E). Integrated analysis of RNA sequencing and *NSUN2* RIP-seq identified several candidate target genes (including *STC1*, *CDCP1*, *FOSL1*, and *Serpine1*) associated with tumor invasion and neuronal interactions (Figure 5F) 22-25. We next validated these targets. M5C-RIP assays demonstrated a marked increase in m5C modification of *CDCP1* and *STC1* upon lactate treatment (Figure 5G). Actinomycin D chase experiments confirmed that lactate enhanced the stability of both transcripts in an *NSUN2*-dependent manner (Figure 5H-I). In contrast, *FOSL1* and *Serpine1*, although identified in the integrated analysis, exhibited no detectable changes in m5C modification or mRNA stability (Figure S6A-B), suggesting they are not bona fide *NSUN2*-m5C targets. To assess whether *NSUN2* is required for the lactate-induced upregulation of *CDCP1* and *STC1*, we examined their transcript and protein levels following *NSUN2* knockdown with or without lactate treatment. Lactate exposure markedly increased both mRNA and protein abundance of *CDCP1* and *STC1*, whereas silencing *NSUN2* reduced their basal expression and attenuated the lactate-induced elevation (Figure 5J-K). We next asked whether *CDCP1* and *STC1* mediate the PNI phenotypes. Findings from Khan, T. *et al.* demonstrated that *CDCP1* promotes the migration and invasion of tumor cells 23. Additionally, Liu *et al.* revealed that *STC1* activates the MAPK/ERK signaling pathway, which plays a crucial role in cell proliferation, migration, and neuronal axon growth 22. Consistent with these findings, our experiments demonstrated that knockdown of *CDCP1* or *STC1* significantly reduced tumor-cell migration and invasion, as well as neurite outgrowth and DRG invasion, whereas exogenous lactate partially restored these phenotypes (Figure 5L-M; Figure S6C-E). Furthermore, cross-linking immunoprecipitation (CLIP) analysis demonstrated that lactate treatment enhanced the selective interaction between *NSUN2* and *CDCP1*/*STC1* mRNAs (Figure 5N). Furthermore, IGV peak analysis revealed that *NSUN2* binding sites were predominantly enriched in the 3' UTR region, which is known to contribute to mRNA stability (Figure 5O) 26. To directly test whether these candidate motifs mediate m5C deposition, we generated wild-type and mutant constructs of *CDCP1* and *STC1* (Figure 5P). MeRIP-qPCR revealed robust m5C enrichment in wild-type constructs, which was markedly reduced in the mutants (Figure 5Q-R). Consistently, actinomycin D chase assays demonstrated that wild-type conferred greater transcript stability than their mutant counterparts (Figure 5S-T).

To further validate the regulatory axis, we examined whether *NSUN2* modulates *CDCP1* and *STC1* expression in an m5C-dependent manner. Methylated RNA immunoprecipitation assays revealed that wild-type *NSUN2* markedly increased m5C enrichment on *CDCP1* and *STC1* transcripts, whereas the catalytic mutant lost this ability (Figure S6F). Consistently, CLIP analysis demonstrated that the *NSUN2* catalytic mutant exhibited markedly reduced association with both *CDCP1* and *STC1* transcripts (Figure S6G), accompanied by a decrease in their mRNA and protein abundance (Figure S6h-i). Decay assays further demonstrated shortened half-lives of *CDCP1* and *STC1* mRNAs in *NSUN2*-mut cells compared with *NSUN2*-WT (Figure S6J-K). Functionally, loss of *NSUN2* catalytic activity impaired tumor-cell migration, invasion, and DRG neurite outgrowth, as shown in transwell and co-culture assays (Figure S6L-N). To further validate the regulatory role of *NSUN2* lactylation in modulating *CDCP1* and *STC1*, we assessed how K692 mutations affect their expression and stability. Compared with *NSUN2*-WT, the lactylation-mimetic K692E mutant markedly elevated the expression of both genes, whereas the non-lactylatable K692R mutant suppressed their expression, with lactate supplementation amplifying these trends (Figure S7A). In keeping with these findings, actinomycin D chase assays showed prolonged half-lives of *CDCP1* and *STC1* mRNAs in the presence of K692E, while K692R accelerated their decay (Figure S7B-C). At the signaling level, *NSUN2* overexpression enhanced ERK phosphorylation and upregulated c-FOS, and these effects were abolished when *STC1* was depleted or blocked by neutralizing antibody (Figure S7D). Functionally, *NSUN2*-driven neurite outgrowth and neural invasion were strictly dependent on *STC1*: silencing or antibody blockade eliminated the neurotropic advantage, whereas forced *STC1* expression partially restored neural invasion under FOS knockdown conditions (Figure S7E-G). Collectively, these results demonstrate that *NSUN2* regulates *CDCP1* and *STC1* mRNA stability in a manner dependent on m5C modification.

### Impact of *NSUN2* lactylation on PNI in pancreatic cancer *in vivo*

**Figure 6:**
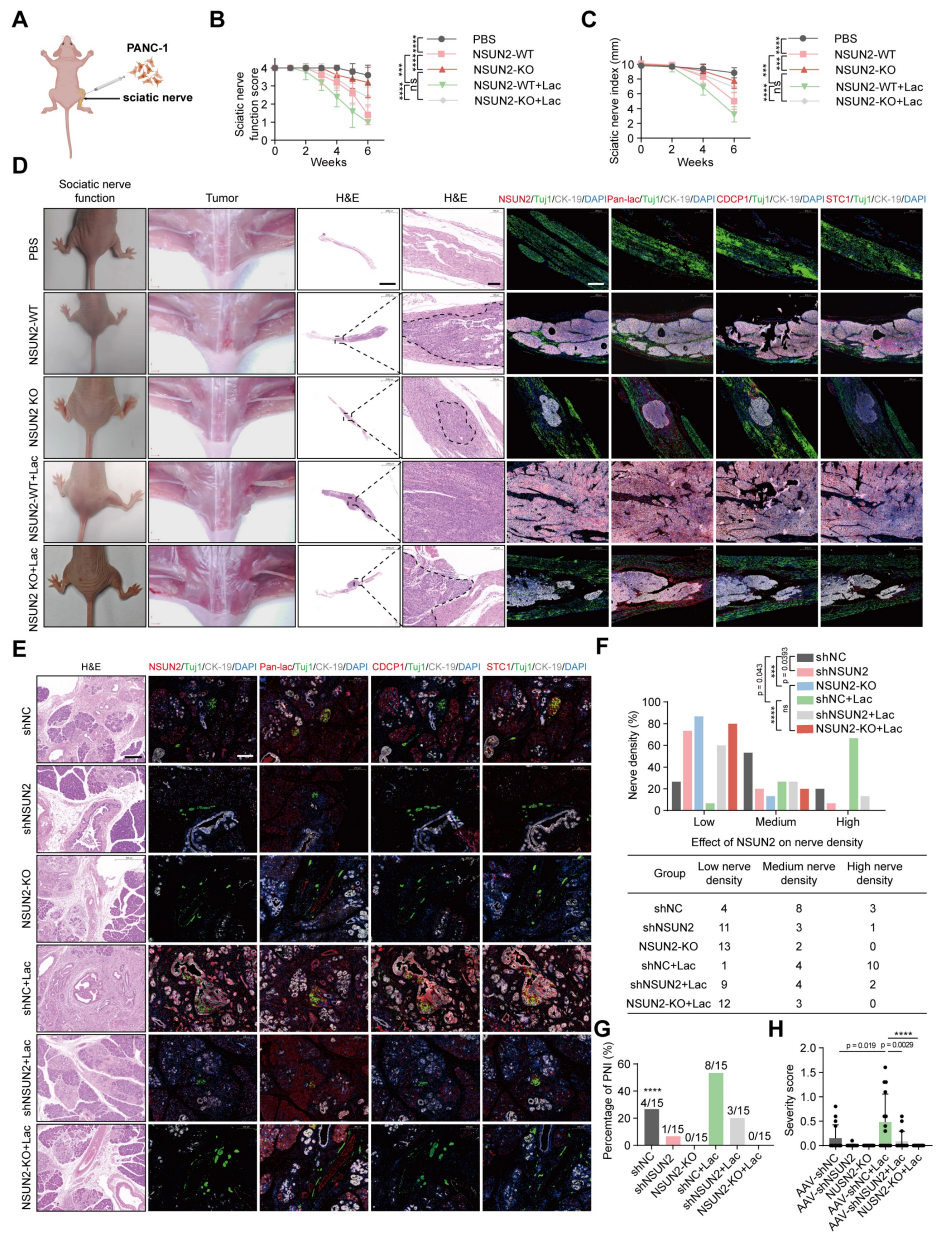
** Impact of *NSUN2* Lactylation on PNI in PDAC *in vivo*. (A)** Schematic diagram of the sciatic nerve invasion model in nude mice. *NSUN2*-knockout PANC-1 cells were generated using the CRISPR/Cas9 system. Wild-type or *NSUN2*-knockout PANC-1 cells were treated with either L-lactate or PBS, followed by inoculation into the sciatic nerve of nude mice. PBS alone served as the control. (n=5 per group). **(B-C)** Sciatic nerve function scores **(B)** and sciatic nerve indexes **(C)** of mice treated as indicated. (n = 5; mean ± SD; Kruskal-Wallis test with Dunn's multiple comparisons test). **(D)** Representative gross morphology, intraoperative views, H&E and mIF images from the sciatic nerve invasion model. mIF demonstrates the expression of Pan-lac, *NSUN2*, *CDCP1*, and *STC1* at the tumor-nerve interface. Dashed lines mark the tumor margins. *Tuj1* serves as a neuronal marker, whereas *CK-19* identifies tumor epithelial cells. Scale bar for H&E, 1000 μm; scale bar for mIF, 200 μm. **(E-H)** KPC mice (LSL-KRASG12D/+; LSL-TP53R172H/+; PDX-1-CRE+/+) were divided into six groups, each receiving orthotopic injections into the pancreas. Two groups were injected with adeno-associated virus (AAV) packaging short hairpin RNA negative control (shNC), while the other two groups were injected with AAV packaging sh*NSUN2*. One week later, control and experimental mice received sodium lactate (1 g/kg) or PBS intraperitoneally, respectively. n = 15. **(E)** H&E and mIF images demonstrate the PNI features in KPC mice and expression of Pan-lac, *NSUN2*, *CDCP1*, and *STC1*. *Tuj1* is used as a neuronal marker, while *CK-19* denotes tumor epithelium. Scale bars: H&E and mIF, 200 μm. **(F)** Quantitative assessment of nerve density (above) and nerve count (below) in KPC mice. (n = 15; Chi-square test). **(G)** PNI frequency in KPC mice. (n = 15; Fisher's exact test). **(H)** Severity score of KPC mice. (n = 15; mean ± SD; one-way ANOVA test). ****P < 0.0001, ***P < 0.001, **P < 0.01, *P < 0.05.

To evaluate the *in vivo* relevance of *NSUN2* lactylation in perineural invasion (PNI), we employed a murine sciatic nerve invasion model. PANC-1 cells with wild-type *NSUN2* or *NSUN2* knockout were pretreated with either L-lactate or PBS and were inoculated into the sciatic nerves of nude mice (Figure 6A). Animals receiving *NSUN2*-WT cells developed progressive ipsilateral hind-limb paralysis and reduced paw span, whereas *NSUN2* depletion markedly attenuated these phenotypes. Exogenous lactate exacerbated functional decline, but this effect was largely abolished in the absence of *NSUN2* (Figure 6B-C). Histological and multiplex immunofluorescence analyses confirmed that *NSUN2* deletion reduced tumor-induced PNI and downregulated *CDCP1* and *STC1*, while lactate supplementation increased pan-lactylation, *NSUN2*, *CDCP1*, and *STC1* expression. Importantly, the loss of *NSUN2* eliminated this lactate-driven induction (Figure 6D, Figure S8A-G).

To better mimic endogenous tumor-nerve interactions, we further utilized KPC mice (LSL-KRAS^G12D/+, LSL-TP53^R172H/+, PDX1-Cre^+/+) that spontaneously develop pancreatic cancer. *In situ* AAV-mediated delivery of sh*NSUN2* efficiently silenced *NSUN2* expression, as verified by both qPCR and immunofluorescence quantification of tumor tissues (Figure S8H-J). In addition, we utilized CRISPR/Cas9-mediated frameshift mutations to achieve site-specific knockout of *NSUN2* in the pancreas of KPC mice, thereby further confirming the functional role of *NSUN2 in vivo* (Figure 6E). One week later, mice were administered systemic lactate or PBS. Histological and multiplex immunofluorescence analyses revealed that *NSUN2* knockdown suppressed *CDCP1* and *STC1* expression and reduced intratumoral nerve density, whereas lactate treatment partially restored these features (Figure 6E; Figure S8K-N).

Notably, the lactate-induced enhancement of perineural invasion and upregulation of *CDCP1* and *STC1* expression were abolished in *NSUN2*-deficient KPC mice (Figure 6E; Figure S8K-N). Biochemical measurements showed that systemic lactate administration significantly elevated intratumoral lactate concentrations, confirming successful delivery and metabolic impact within the tumor microenvironment (Figure S8O). Consistently, quantification showed a reduced incidence and severity of PNI upon *NSUN2* depletion, with lactate again exerting only partial rescue effects (Figure 6F-G). To more rigorously assess neural involvement, tumor sections were stained with the neuronal marker β3-tubulin and independently scored by professional pathologist. PNI severity score = [(no. of nerves / n0 × 'score 0') + (n1 × 'score 1' / perineural invasion) + (n2 × 'score 2' / endoneural invasion)] / total no. of nerves, where n0, n1 and n2 denote the number of nerves without invasion, with perineural invasion, and with endoneural invasion, respectively 27. Using this approach, we observed that lactate treatment significantly increased PNI severity, whereas *NSUN2* silencing reduced it (Figure 6H). Collectively, these findings demonstrated that *NSUN2* lactylation enhances perineural invasion and tumor nerve interactions in pancreatic cancer models.

### Association of *NSUN2*/*CDCP1*/*STC1* with PNI and unfavorable prognosis in PDAC

**Figure 7:**
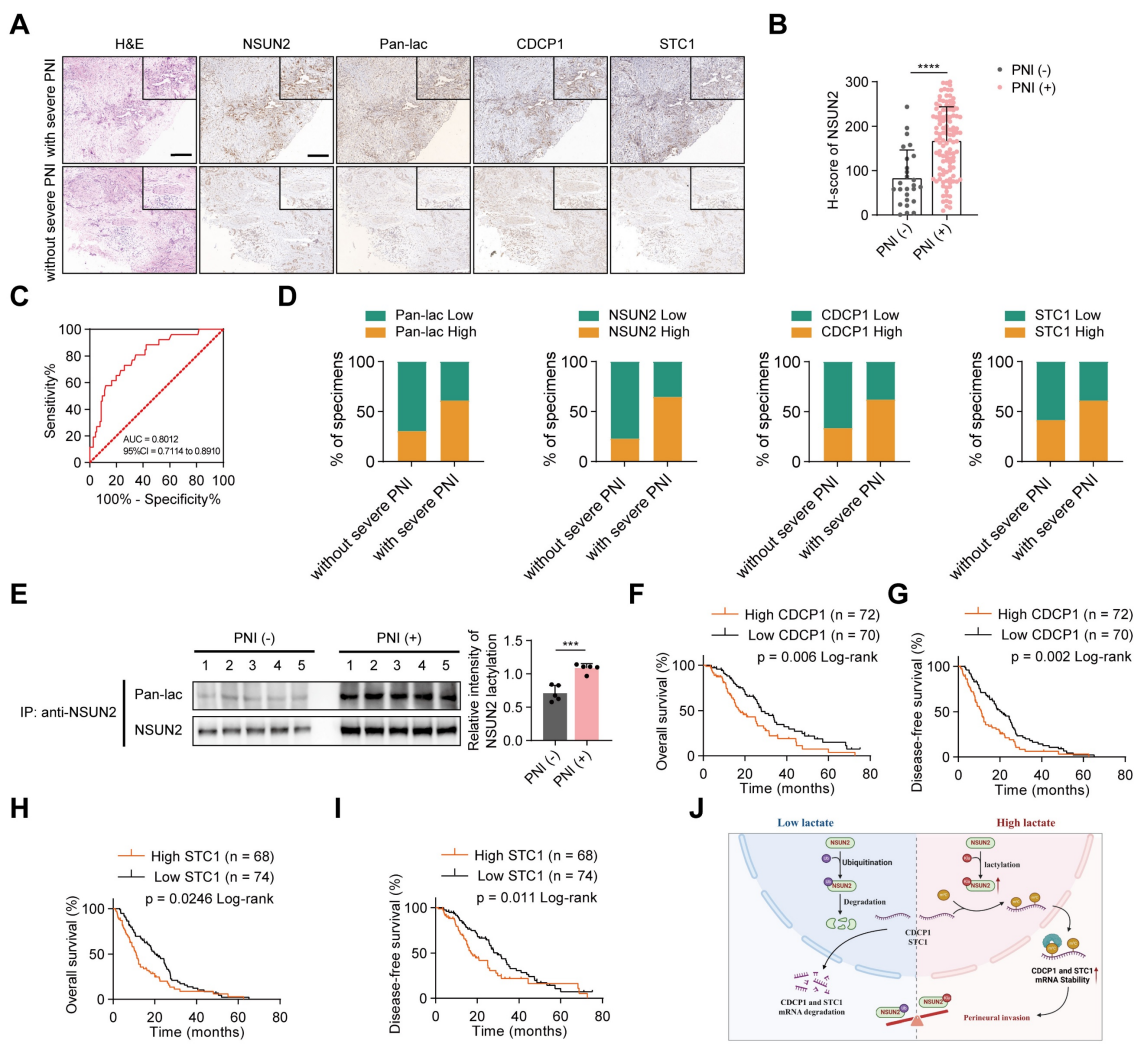
** Association of *NSUN2*/*CDCP1*/*STC1* with PNI and Unfavorable Prognosis in PDAC. (A)** H&E and IHC images showing Pan-lac, *NSUN2*, *CDCP1*, *STC1* expression in PDAC specimens (n = 142). Scale bars, 200 μm. **(B)** H-score of *NSUN2* expression in PDAC patients with or without severe PNI. **(C)** ROC curve analysis based on *NSUN2* H-scores to further evaluate its predictive ability for PNI risk. **(D)** Proportional distribution of Pan-lac, *NSUN2*, *CDCP1*, and *STC1* expression in PDAC samples grouped by PNI severity (with or without severe PNI). **(E)** Lactylation of *NSUN2* in PNI(-) or PNI(+) pancreatic cancer tissues (left) and quantification of *NSUN2* lactylation intensity normalized to *NSUN2* (n = 3; unpaired t test; mean ± SD). **(F-I)** Kaplan-Meier survival curves showing OS and DFS in the PDAC cohort stratified by *CDCP1* **(F-G)** and *STC1* **(H-I)** level (high vs low). **(J)** Diagram summarizing the proposed mechanism through which a lactate-enriched tumor microenvironment facilitates pancreatic cancer PNI by upregulating tumor cells *NSUN2* lactylation and transcriptionally activating the m5C modification of neural invasion—related genes.

To investigate the clinical significance of *NSUN2* lactylation in PDAC, we assessed their expression via immunohistochemistry (IHC) in tissue samples from 142 PDAC patients, categorized by the presence or absence of severe PNI (Figure 7A). To more precisely assess the clinical relevance of *NSUN2*, we quantified IHC staining using the H-score method, and the H-score was calculated as: (percentage of weak staining) + 2 × (percentage of moderate staining) + 3 × (percentage of strong staining), yielding a total score of 0-300 28. When applied to patient samples, this scoring approach revealed that PNI (+) cases exhibited significantly higher *NSUN2* levels than PNI (-) cases (Figure 7B). To further evaluate the diagnostic performance, we conducted ROC curve analysis, which demonstrated that *NSUN2* expression robustly discriminated PNI (+) from PNI (-) tumors, underscoring its predictive value for PNI risk (Figure 7C). Elevated levels of lactylation and *NSUN2*-related proteins were detected in PNI-high tumors compared with PNI-low cases (Figure 7D). To clarify the potential clinical implications of *NSUN2* lactylation, we performed co-immunoprecipitation (Co-IP) followed by western blotting on protein extracts from pancreatic tumor tissues. The results revealed that tumors from PNI (+) patients exhibited markedly higher levels of lactylated *NSUN2* compared with those from PNI (-) patients (Figure 7E), providing direct clinical evidence that *NSUN2* lactylation is associated with perineural invasion. Kaplan-Meier survival analysis demonstrated that a high *CDCP1* and *STC1* expression was associated with shorter overall survival (OS) and disease-free survival (DFS) (Figure 7F-I). In addition, we performed comprehensive univariate and multivariate Cox regression analyses for both overall survival (OS) and disease-free survival (DFS) in our cohort of 142 PDAC patients. The results demonstrated that PNI was significantly associated with poor prognosis in the univariate model, and importantly, this association remained robust after adjusting for TNM stage and tumor differentiation (Table S4-S5). These findings clearly indicate that the prognostic value of PNI extends beyond established clinical variables and underscore its importance as an independent marker of patient outcome in PDAC. Consistent with these clinical correlations, mechanistic integration of our findings supports a model in which lactylation serves as a pivotal switch controlling *NSUN2* stability and downstream oncogenic signaling (Figure 7J). Under low-lactate conditions, *NSUN2* undergoes ubiquitination and degradation, leading to destabilization and decay of *CDCP1* and *STC1* transcripts, thereby attenuating PNI. In contrast, high lactate levels promote *NSUN2* lactylation, which prevents ubiquitination-mediated degradation, enhances *NSUN2* stability, and facilitates selective binding to *CDCP1* and *STC1* mRNAs. Collectively, our findings suggest that lactylation of *NSUN2* may serve as a biomarker for PNI and a potential therapeutic target in PDAC.

## Discussion

While substantial progress has been made in unraveling the complex molecular and signaling interactions involved in PNI, the specific mechanisms by which lactate metabolism drives its progression remain largely unclear. Recent research by Li *et al.* demonstrated that cancer-associated fibroblasts (CAFs) create a lactate-rich microenvironment, which drives histone H3K18 lactylation and consequently promotes the perineural invasion (PNI) of pancreatic cancer 29. In addition, Chen *et al.* showed that lactate-induced lactylation of *NSUN2* enhances its binding affinity to ENO1 mRNA and promotes m5C-dependent stabilization, thereby accelerating colorectal cancer progression 7. These findings collectively highlight the critical roles of both histone and non-histone protein lactylation in promoting tumor progression. Our study demonstrates that patients with severe PNI exhibit markedly elevated lactate and global lactylation levels, which are associated with poor prognosis, identifying *NSUN2* as a key mediator in lactylation-induced PNI. Mechanistically, lactate accumulation promotes lactylation of *NSUN2* at K692, preventing its degradation and enhancing *CDCP1* and *STC1* mRNA stability through lactylation modification, and inhibition of *NSUN2* or its lactylation significantly reduces neural invasion in a KPC mouse model. Our study reveals that lactylation-induced *NSUN2*-mediated RNA m5C modification is a crucial driver of PNI in PDAC, highlighting *NSUN2* inhibition as a novel and promising therapeutic strategy for addressing this aggressive feature of pancreatic cancer.

Hypoxic tumor cells predominantly rely on glucose for glycolytic energy production, leading to the release of lactic acid, which establishes a lactate gradient that mirrors the tumor's oxygen distribution 30. Initial studies on metabolites in human cervical cancer 31 and head and neck squamous cell carcinomas 32 suggest a link between the levels of lactate in tumor tissue and the likelihood of metastatic spread. Yu *et al.* recently reported that reducing lactate levels in the tumor microenvironment can effectively alleviate immunosuppression and boost immune responses, offering a promising approach for pancreatic cancer treatment 33. Li *et al.* revealed that enhanced glycolysis, driven by glutamate-induced m6A modification of HK2 mRNA, plays a crucial role in promoting PNI in PDAC, highlighting the significant impact of metabolic regulation on PNI 34. Nevertheless, the correlation between lactate levels and the extent of PNI in PDAC has not been elucidated. Our study is the first to establish this connection, suggesting a critical role for lactate in modulating PNI, thereby unveiling a previously uncharted dimension of tumor pathophysiology. Lactylation, a key and widespread post-translational protein modification, has recently been shown to be highly expressed in PDAC and is associated with poor patient prognosis 35. Wang *et al.* recently demonstrated that PYCR1 regulates lactate metabolism via histone lactylation to modulate IRS1 expression, thereby promoting liver cancer metastasis 36. Our research found that patients with advanced PNI show significantly elevated pan-lactylation, highlighting a crucial mechanism of metabolic-epigenetic coupling within the PNI microenvironment in PDAC.

Growing evidence has established a link between aberrant m5C modifications and a range of diseases, including cancers, heart disease, neurodegenerative disorders, and chronic inflammation 8,9,37-41. We recently found that CAFs enhance pancreatic cancer cell PNI by modulating the function of YBX1, an m5C reader, enabling it to selectively recognize and stabilize PNI-related mRNAs 10. Notably, we also observed a correlation between increased PNI and elevated m5C levels, though the underlying mechanism remains to be explored. Recent studies have highlighted the critical role of lactate in regulating RNA modifications, particularly through lactylation-driven mechanisms 42. Xiong *et al.* demonstrated that lactate induces the expression and activity of the RNA methyltransferase METTL3, enhancing the immunosuppressive functions of tumor-infiltrating myeloid cells and promoting immune evasion in tumors 43. Similarly, Yu *et al.* showed that histone lactylation accelerates tumorigenesis by activating the m6A reader YTHDF2 44, while Gu *et al.* further demonstrated that lactylation activates ALKBH3, modulating m1A modification, thereby highlighting the close interplay between lactylation and RNA methylation in tumor progression 45. In our study, we for the first time connected lactylation with m5C RNA modification, uncovering a novel regulatory axis in tumor metastasis and advancing the understanding of how metabolic and epigenetic modifications interact.

A previous study reported that the expression of the m5C methyltransferase NOP2/Sun RNA methyltransferase family member 2 (*NSUN2*) was aberrantly elevated in pancreatic tumors 46. The role of lactylation in regulating *NSUN2* expression in pancreatic cancer remains unclear. Recent studies have highlighted the role of *NSUN2* upregulation and its RNA m5C modification in Cr(VI)-induced lung tumor progression, where *NSUN2* expression is transcriptionally activated by EP300 via histone acetylation (H3K27ac) 47. Additionally, in colorectal cancer cells, lactate accumulation promotes *NSUN2* transcription through histone H3K18 lactylation, which in turn induces lactylation of *NSUN2* at Lys356, crucial for binding to specific RNAs and facilitating the m5C modification of ENO1 mRNA 7. In contrast to previous studies, our research found that lactate does not upregulate the mRNA expression of *NSUN2* in pancreatic cancer cells. However, lactylation of *NSUN2* inhibits its ubiquitination and degradation by disrupting its interaction with *STUB1*, thereby enhancing its stability in the presence of lactate. This suggests that lactylation modification may exert heterogeneous regulatory effects across different diseases and highlights the functional diversity of lactylation in modulating key protein modifications. Recent studies have illuminated the complex post-translational regulation of *NSUN2*, highlighting its diverse functional modifications. Zhang *et al.* identified *NSUN2* as a target of the E3 ubiquitin ligase *STUB1*, which mediates its ubiquitination and degradation during hepatocyte ferroptosis 19. Additionally, SUMO-2/3 has been shown to stabilize *NSUN2* and facilitate its nuclear transport in gastric cancer 48, while glucose-induced oligomerization and activation of *NSUN2* promote TREX2 expression, suppressing cGAS/STING activation to regulate oncogenic activity in cancer cells 49. We elucidated that lactylation at *NSUN2*-K692 inhibits its ubiquitination and promotes PNI in pancreatic cancer. RIP-seq shows that *NSUN2* binds chiefly within coding exons, with fewer but notable peaks in 3' UTRs and very few in 5' UTRs. Lactate slightly amplifies the 3' UTR fraction without changing this hierarchy. This distribution pattern is consistent with the RIP-seq results of *NSUN2* reported by Wenlan Yang *et al.*, which showed that in Th17 cells, *NSUN2*-binding peaks were primarily located in the CDS region of mRNA transcripts, followed by the 3' UTR 50. Through comprehensive analysis of *NSUN2* RIP-Seq and RNA-Seq under L-lactate treatment, we identified PNI-associated genes, *CDCP1* and *STC1*, as key targets. Further MeRIP-PCR analysis revealed that *NSUN2* primarily enhances the m5C modification on *CDCP1* and *STC1*, thereby stabilizing their mRNA. While Chen *et al.* shown that lactate binds to *NSUN2*-K356 to facilitate the capture of m5C-modified RNAs in colorectal cancer 7, whether lactylation at *NSUN2*-K692 similarly aids in the m5C modification of *CDCP1* and *STC1* remains an intriguing avenue for further investigation. Our study unveils the novel role of *NSUN2* lactylation at K692 in regulating m5C modifications of *CDCP1* and *STC1*, emphasizing the distinctive role of lactylation in modulating PNI and highlighting its potential as a therapeutic target.

While previous studies have demonstrated that both histone and non-histone lactylation contribute to pancreatic cancer invasion and metastasis 17,51, and that lactylation can regulate tumor progression by influencing methylation modifications 52, our study presents a novel perspective by uncovering the cooperative role of lactylation and m5C methylation in perineural invasion (PNI) of pancreatic cancer. Specifically, we reveal that metabolic activation leads to intracellular lactate accumulation, which selectively induces lactylation of *NSUN2*, the critical m5C writer enzyme. This modification enhances *NSUN2* protein stability, thereby increasing m5C modification-mediated stabilization of key PNI-related genes. The resulting upregulation of their post-transcriptional expression creates a previously unrecognized regulatory axis between protein lactylation and RNA methylation, which plays a crucial role in PNI progression. By elucidating this precise regulatory interplay between protein and RNA modifications, our findings expand the understanding of metabolic-epigenetic crosstalk in pancreatic cancer and offer novel insights into targeted therapeutic strategies aimed at disrupting specific modification pathways. This study provides a foundation for future research into metabolic reprogramming-driven epigenetic regulation in aggressive tumor behaviors, including PNI.

In conclusion, our study is the first to uncover the cooperative role of epigenetic modifications in governing PNI progression in pancreatic cancer. Our findings demonstrate that major PNI-related genes are tightly regulated through *NSUN2*-mediated m5C modification, which is driven by lactylation of *NSUN2*. Our findings highlight the potential of targeting *NSUN2* lactylation and inhibiting m5C modification as promising strategies to prevent PNI and improve therapeutic outcomes in pancreatic cancer.

## Supplementary Material

Supplementary figures and tables.
