# Supplementary material for: Lactylation-driven NSUN2-mediated RNA m5C modification promotes perineural invasion in pancreatic cancer
Source: Theranostics. 2026 Jan 1;16(4):1782–803. doi: 10.7150/thno.122294 (PMC12680535; doi:10.7150/thno.122294)
Supplement: Supplementary file 1 — Supplementary figures and tables. [file thnov16p1782s1.pdf]

Table of Contents

Figure S1. PNI in PDAC patients correlates with lactylation of primary cells. 2

Figure S2. Lactate promotes proliferation, migration and neural tropism of PDAC cells. 4

Figure S3. Effect of *NSUN2* in PNI of pancreatic cancer cells. 6

Figure S4. Quantification of *NSUN2* protein and lactylation under various treatments. 8

Figure S5. *NSUN2*-K692 mutation is associated with PNI. 9

Figure S6. *CDCP1*/*STC1* is associated with PNI. 12

Figure S7. *NSUN2* K692 lactylation stabilizes *CDCP1*/*STC1* and promotes PNI. 14

Figure S8. Quantification of immunofluorescence in vivo experiments related to PNI. 16

Table S1. Primers, probes and oligonucleotides used in the study. 18

Table S2. Clinical and pathologic variables 20

Table S3. Antibody and Kit 21

Table S4 Univariate and multivariate analysis of Overall Survival (OS) in PDAC patients (n = 142) 22

Table S5 Univariate and multivariate analysis of Disease-free Survival (DFS) in PDAC patients (n = 142) 23

Figure S1. PNI in PDAC patients correlates with lactylation of primary cells.

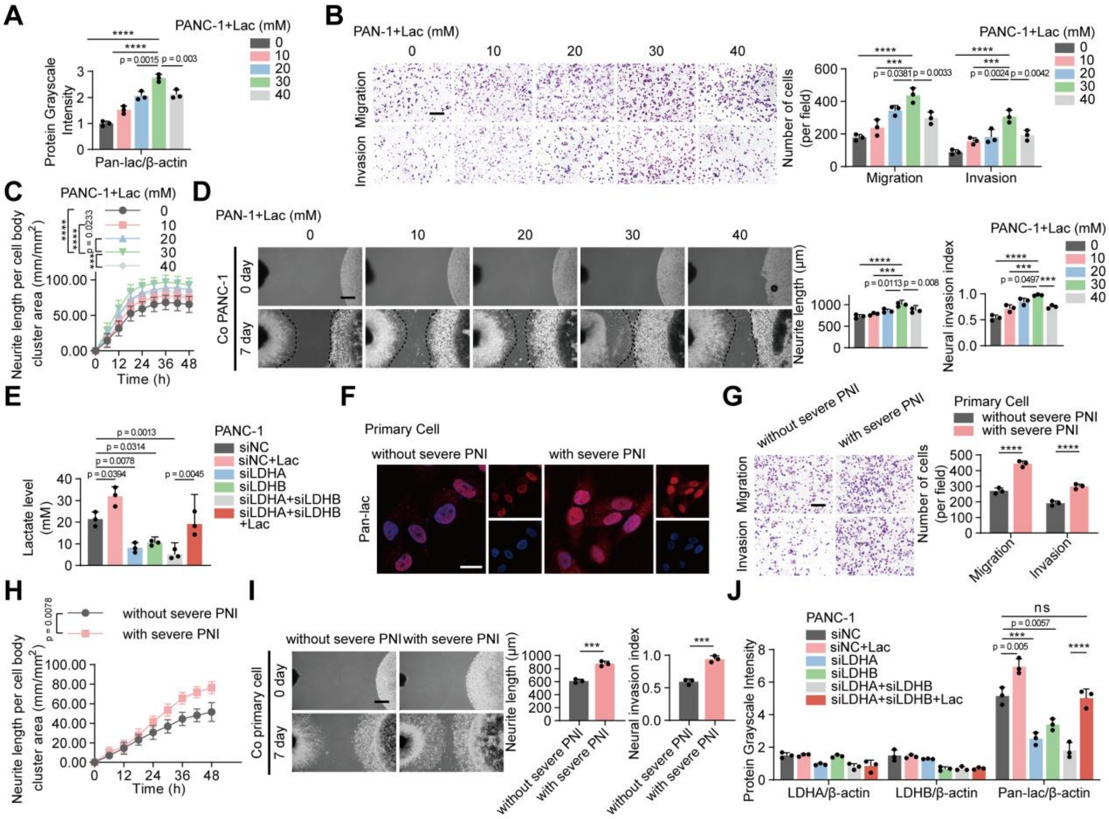

Figure S1. PNI in PDAC patients correlates with lactylation of primary cells.

(A) Quantification of lactylation intensity normalized to *β-actin*. **(B-D)** PANC-1 cells treated with varying concentrations of lactate (0, 10, 20, 30, and 40 mM) for subsequent experiments. **(B)** PANC-1 Transwell migration/invasion: image panels (left) and measurements (right). Scale bar, 200 μm. **(C)** Neurite outgrowth was quantified under the indicated conditions in the Transwell co-culture model. Phase-contrast images were obtained at 6 h intervals. **(D)** (left) Representative fields from DRG co-cultures with tumor cells. (right) Summary statistics for tumor neurite invasion toward DRG. The black dashed line on the left indicates the growth boundary of the DRG, which on the right marks the growth boundary of PANC-1 cells. Scale bar, 500 μm. **(E)** Measurement of lactate concentration in PANC-1 cells after *LDHA* and *LDHB* silencing and addition of L-lactate. **(F)** Immunofluorescence staining for Pan-lac in primary tumor cells isolated from patients grouped by PNI severity. Scale bar, 20 μm. **(G-I)** Primary tumor cells were isolated from patients grouped by PNI severity and were used for subsequent experiments. **(G)** Primary tumor cells Transwell migration/invasion: image panels (left) and measurements (right). Scale bar, 200 μm. **(H)** Neurite outgrowth was quantified under the indicated conditions in the Transwell co-culture model. Phase-contrast images were obtained at 6 h intervals. **(I)** (left) Representative fields from DRG co-cultures with tumor cells. (right) Summary statistics for tumor neurite invasion toward DRG. Scale bar, 500 μm. **(J)** Densitometric analysis of protein lactylation levels in PANC-1 cells following *LDHA* and *LDHB* knockdown and L-lactate supplementation, as determined by immunoblot. Each experiment was performed independently in triplicate, and all quantitative results are presented as mean ± SD. Statistical tests used for each panel were as follows: **(A, B, D, E)** one-way ANOVA; **(C)** two-way ANOVA; **(G, I)** unpaired t test; **(H)** paired t test. * for $P \leq 0.05$, ** for $P \leq 0.01$, *** for $P \leq 0.001$ and **** for $P \leq 0.0001$.

Figure S2. Lactate promotes proliferation, migration and neural tropism of PDAC cells.

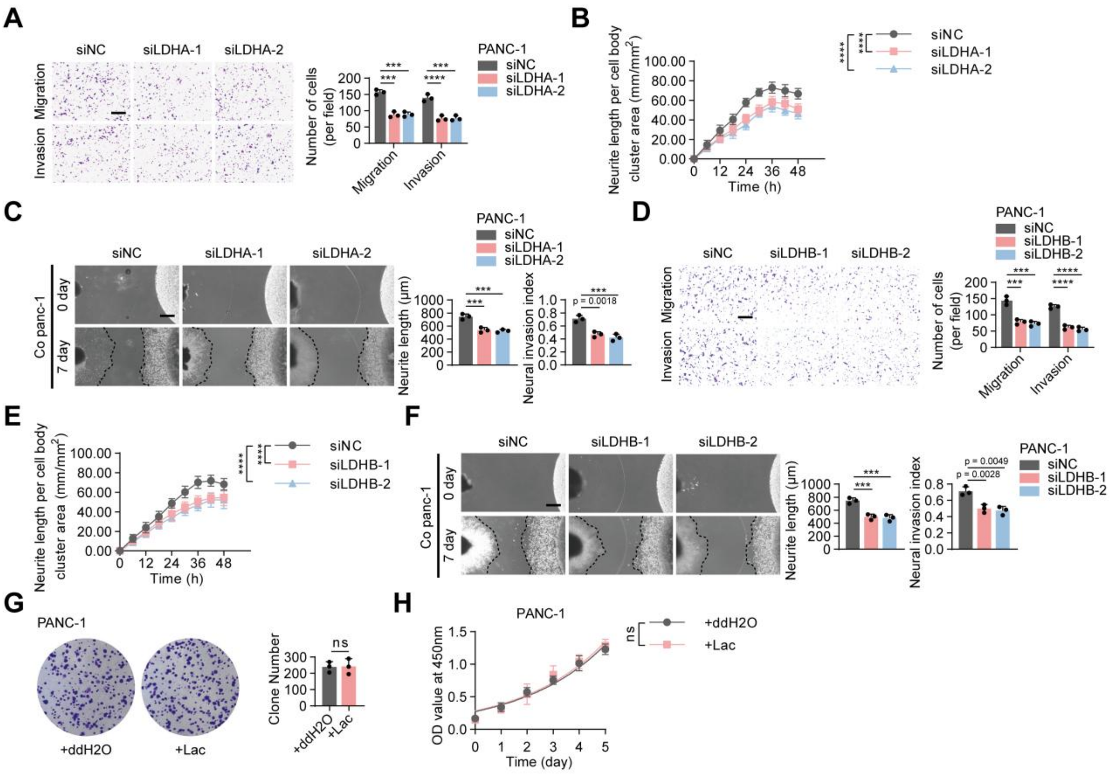

Figure S2. Lactate promotes proliferation, migration and neural tropism of PDAC cells.

(A-C) PANC-1 cells expressing *LDHA* siRNA or siNC were used for subsequent experiments. **(A)** PANC-1 Transwell migration/invasion: image panels (left) and measurements (right). Scale bar, 200 μm. **(B)** Neurite outgrowth was quantified under the indicated conditions in the Transwell co-culture model. Phase-contrast images were obtained at 6 h intervals. **(C)** (left) Representative fields from DRG co-cultures with tumor cells. (right) Summary statistics for tumor neurite invasion toward DRG. Scale bar, 500 μm. **(D-F)** PANC-1 cells expressing *LDHB* siRNA or siNC were used for subsequent experiments. **(D)** PANC-1 Transwell migration/invasion: image panels (left) and measurements (right). Scale bar, 200 μm. **(E)** Neurite outgrowth was quantified under the indicated conditions in the Transwell co-culture model. Phase-contrast images were obtained at 6 h intervals. **(F)** (left) Representative fields from DRG co-cultures with tumor cells. (right) Summary statistics for tumor neurite invasion toward DRG. Scale bar, 500 μm. **(G)** Representative images and quantitative analysis of colony formation in PANC-1 cells treated with ddH_2O or L-lactate. **(H)** CCK-8 assay showing the proliferation of PANC-1 cells exposed to ddH_2O or L-lactate treatment over a period of 5 days. Each experiment was performed independently in triplicate, and all quantitative results are presented as mean ± SD. Statistical tests used for each panel were as follows: **(A-D, F)** one-way ANOVA; **(E)** two-way ANOVA; **(G)** unpaired t test; **(H)** paired t test. * for $P \leq 0.05$, ** for $P \leq 0.01$, *** for $P \leq 0.001$ and **** for $P \leq 0.0001$.

Figure S3. Effect of *NSUN2* in PNI of pancreatic cancer cells.

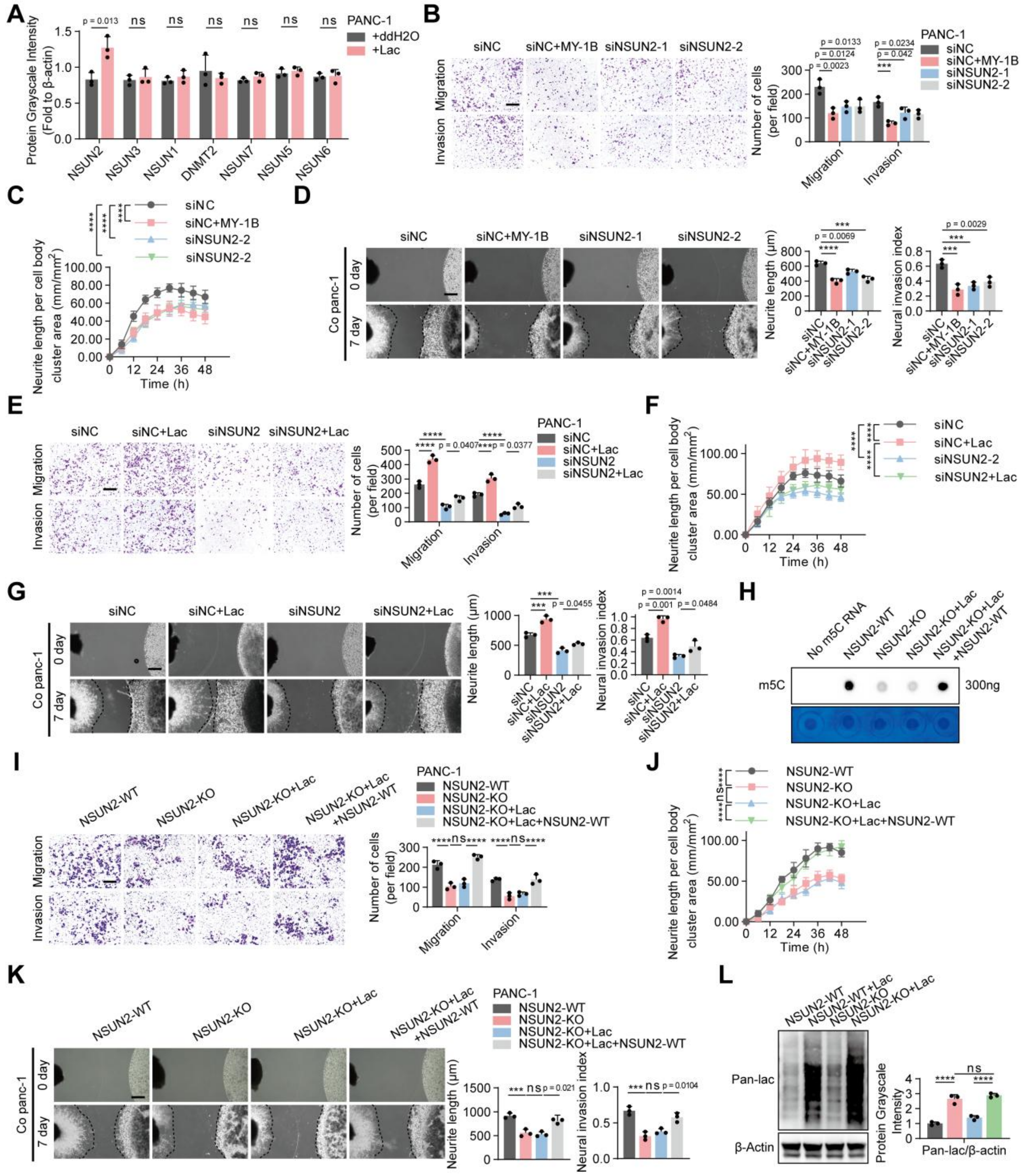

Figure S3. Effect of *NSUN2* in PNI of pancreatic cancer cells.

(A) Densitometric analysis of *NSUN1*, *NSUN2*, *NSUN3*, *NSUN5*, *NSUN6*, *NSUN7* and *DNMT2* protein expression levels in PANC-1 cells with or without L-lactate treatment by Western blot. **(B-D)** PANC-1 cells expressing *NSUN2* siRNA or siNC, or treatment with the *NSUN2* small-molecule inhibitor MY-1B for subsequent experiments. **(B)** PANC-1 Transwell migration/invasion: image panels (left) and measurements (right). Scale bar, 200 μm. **(C)** Neurite outgrowth was quantified under the indicated conditions in the Transwell co-culture model. Phase-contrast images were obtained at 6 h intervals. **(D)** (left) Representative fields from DRG co-cultures with tumor cells. (right) Summary statistics for tumor neurite invasion toward DRG. Scale bar, 500 μm. **(E-G)** PANC-1 cells expressing siNC or *NSUN2*-specific siRNA, followed by treatment with or without L-lactate. **(E)** PANC-1 Transwell migration/invasion: image panels (left) and measurements (right). Scale bar, 200 μm. **(F)** Neurite outgrowth was quantified under the indicated conditions in the Transwell co-culture model. Phase-contrast images were obtained at 6 h intervals. **(G)** (left) Representative fields from DRG co-cultures with tumor cells. (right) Summary statistics for tumor neurite invasion toward DRG. Scale bar, 500 μm. **(H)** m5C dot blot analysis of m^{5}C levels in RNA extracted from PANC-1 cells. Methylene blue staining (below) was used to detect input RNA, while the intensity of the dot blot signal (above) represents the level of m5C modification. **(I)** PANC-1 Transwell migration/invasion: image panels (left) and measurements (right). Scale bar, 200 μm. **(J)** Neurite outgrowth was quantified under the indicated conditions in the Transwell co-culture model. Phase-contrast images were obtained at 6 h intervals. **(K)** (left) Representative fields from DRG co-cultures with tumor cells. (right) Summary statistics for tumor neurite invasion toward DRG. Scale bar, 500 μm. **(L)** Lactylation modification levels were detected in PANC-1 cells by Western blot. Each experiment was performed independently in triplicate, and all quantitative results are presented as mean ± SD. Statistical tests used for each panel were as follows: **(A, B, D, E, G, I, K)** one-way ANOVA; **(C, F, J)** two-way ANOVA; **(L)** unpaired t test. * for $P \leq 0.05$, ** for $P \leq 0.01$, *** for $P \leq 0.001$ and **** for $P \leq 0.0001$.

Figure S4. Quantification of *NSUN2* protein and lactylation under various treatments.

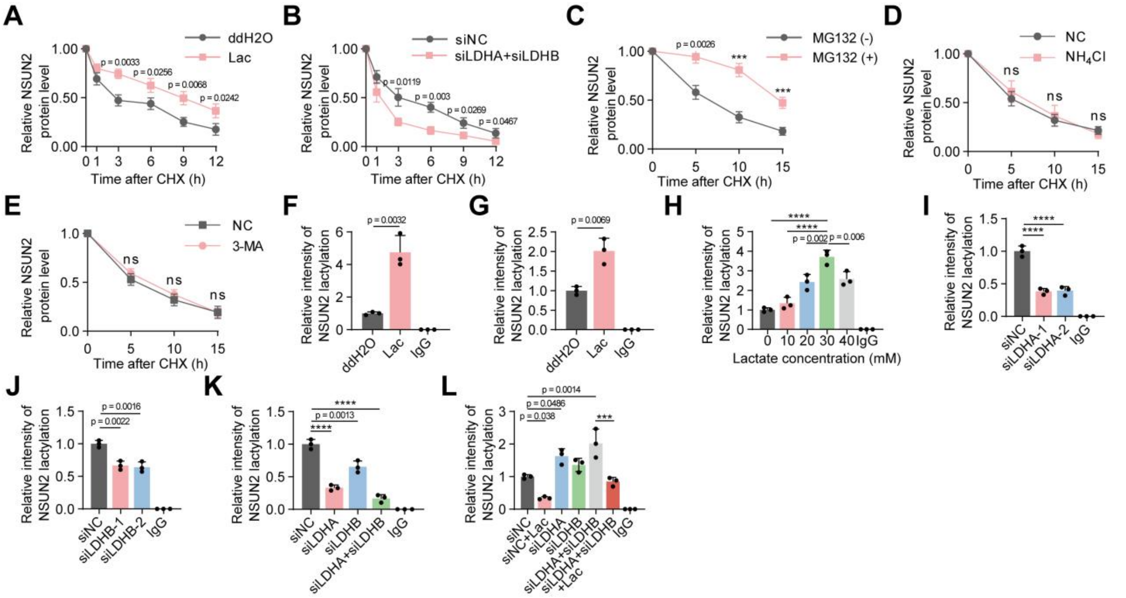

Figure S4. Quantification of *NSUN2* protein and lactylation under various treatments.

(A) Quantitative analysis of *NSUN2* protein expression levels at different time points under ddH2O or L-lactate treatment. **(B)** Quantitative analysis of *NSUN2* protein expression levels at different time points under transfection of siNC or *LDHA*/B siRNAs treatment. **(C)** Quantitative analysis of *NSUN2* protein expression levels at different time points with or without MG132 treatment. **(D-E)** Quantitative analysis of *NSUN2* protein expression levels at different time points under NH_4Cl **(D)** or 3-MA **(E)** treatment. **(F-G)** Quantification of *NSUN2* lactylation intensity normalized to Flag-*NSUN2* in 293T cells **(F)** or PANC-1 cells **(G)**. **(H)** Quantification of *NSUN2* lactylation intensity normalized to Flag-*NSUN2* under different lactate concentration treatment. **(I-L)** Quantification of *NSUN2* lactylation intensity normalized to Flag-*NSUN2* expressing siNC or *LDHA*/B siRNAs exposed to ddH2O or L-lactate treatment. Each experiment was performed independently in triplicate, and all quantitative results are presented as mean ± SD. Statistical tests used for each panel were as follows: **(A-G)** unpaired t test; **(H-L)** one-way ANOVA. * for $P \leq 0.05$, ** for $P \leq 0.01$, *** for $P \leq 0.001$ and **** for $P \leq 0.0001$.

106 **Figure S5. NSUN2-K692 mutation is associated with PNI.**

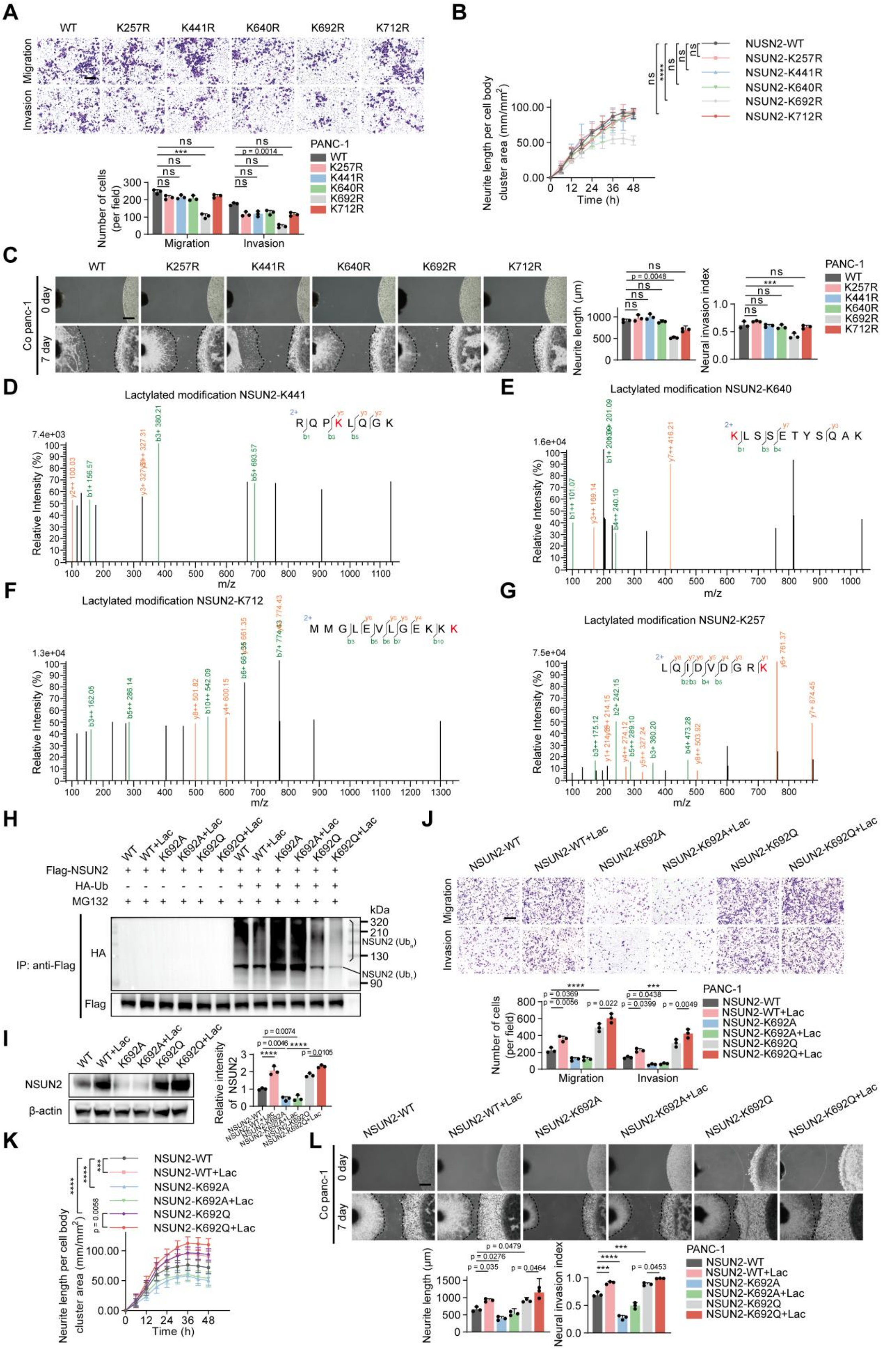

107

Figure S5. *NSUN2*-K692 mutation is associated with PNI.

(A-C) PANC-1 cells expressing indicated *NSUN2* site mutations plasmids were used for subsequent experiments. **(A)** PANC-1 Transwell migration/invasion: image panels (above) and measurements (below). Scale bar, 200 μm. **(B)** Neurite outgrowth was quantified under the indicated conditions in the Transwell co-culture model. Phase-contrast images were obtained at 6 h intervals. **(C)** (left) Representative fields from DRG co-cultures with tumor cells. (right) Summary statistics for tumor neurite invasion toward DRG. Scale bar, 500 μm. **(D-G)** Lactylation sites include **(D)** K441, **(E)** K640, **(F)** K712 and **(G)** K257. **(H-K)** *NSUN2*-K692 mutant PANC-1 cells were generated using the CRISPR / Cas9 approach. Residue K692 was replaced by alanine (K692A) or glutamine (K692Q). **(H)** Lysates from treated PANC-1 cells were SDS-pretreated, immunoprecipitated with anti-FLAG, and probed for *NSUN2* ubiquitination by Western blot. The line labeled '*NSUN2* (Ub1)' indicates mono-ubiquitinated *NSUN2*, while the bracket labeled '*NSUN2* (Ubn)' marks high molecular weight polyubiquitinated *NSUN2* species. **(I)** (Left) *NSUN2* levels were detected in PANC-1 cells by Western blot. (Right) Quantification of *NSUN2* intensity normalized to *β-actin*. **(J)** PANC-1 Transwell migration/invasion: image panels (above) and measurements (below). Scale bar, 200 μm. **(K)** Neurite outgrowth was quantified under the indicated conditions in the Transwell co-culture model. Phase-contrast images were obtained at 6 h intervals. **(L)** (top) Representative fields from DRG co-cultures with tumor cells. (bottom) Summary statistics for tumor neurite invasion toward DRG. Scale bar, 500 μm. Each experiment was performed independently in triplicate, and all quantitative results are presented as mean ± SD. Statistical tests used for each panel were as follows: **(A, C, I-L)** one-way ANOVA; **(B)** two-way ANOVA. * for $P \leq 0.05$, ** for $P \leq 0.01$, *** for $P \leq 0.001$ and **** for $P \leq 0.0001$.

Figure S6. *CDCP1*/*STC1* is associated with PNI.

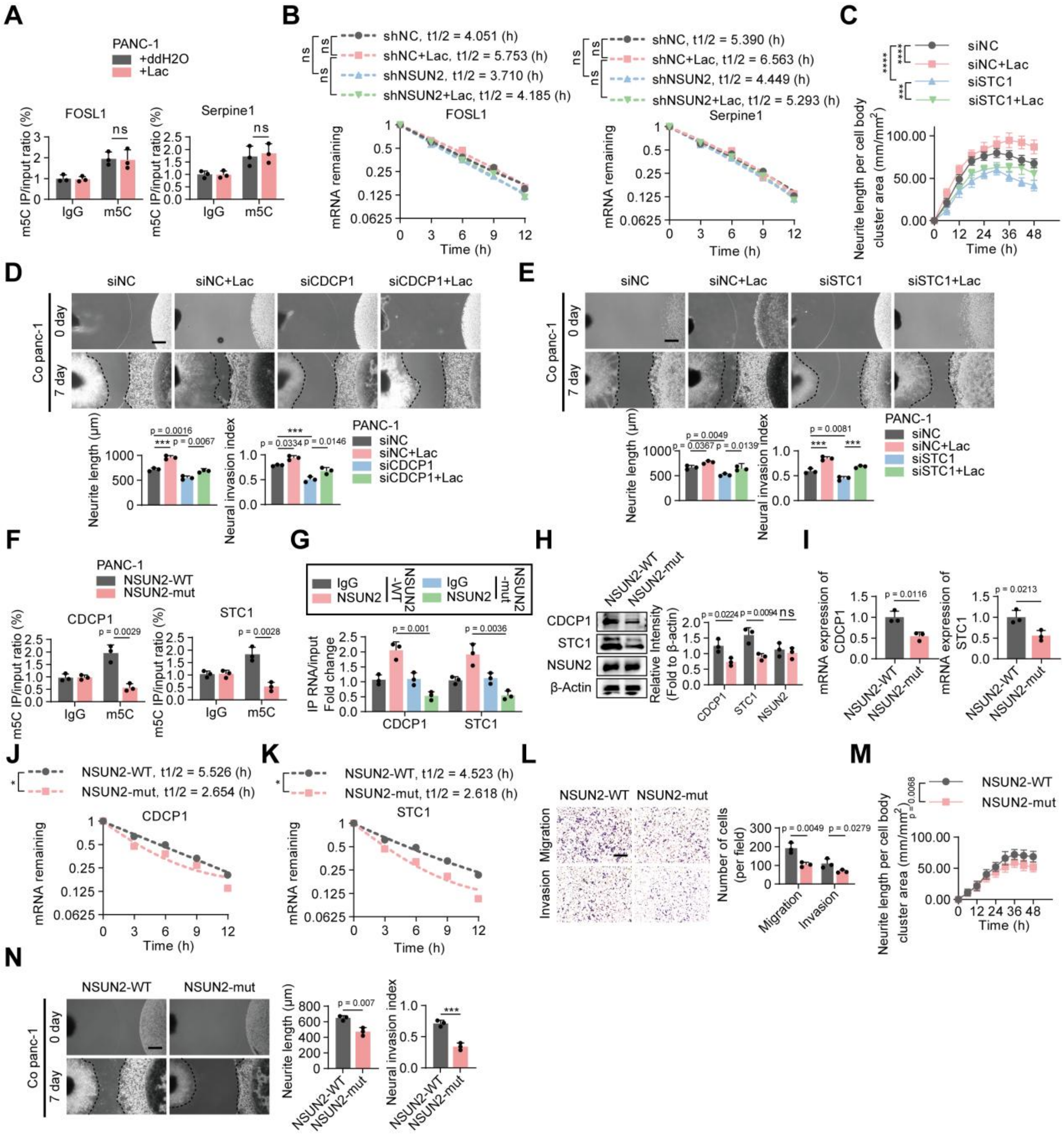

Figure S6. *CDCP1*/*STC1* is associated with PNI.

(A) MeRIP-qPCR confirmed m5C enrichment of *FOSL1* and *Serpine1* transcripts in PANC-1 cells ± L-lactate. **(B)** Actinomycin D assay to assess the half-life of *FOSL1* and *Serpine1* mRNA in PANC-1 cells expressing shNC or *NSUN2*-specific shRNA, followed by exposing to ddH2O or L-lactate treatment. **(C)** Neurite outgrowth was quantified under the indicated conditions in the Transwell co-culture model. Phase-contrast images were obtained at 6 h intervals. **(D)** (top) Representative fields from DRG co-cultures with tumor cells. (bottom) Summary statistics for tumor neurite invasion toward DRG. Scale bar, 500 μm. **(E)** (top) Representative fields from DRG co-cultures with tumor cells. (bottom) Summary statistics for tumor neurite invasion toward DRG. Scale bar, 500 μm. **(F-N)** PANC-1 cells were expressing vector or *NSUN2* methylation domain mutant plasmid. **(F)** MeRIP-qPCR analysis of *CDCP1* and *STC1* mRNA in the m5C peak regions in tumor cells expressing either wild-type *NSUN2* or *NSUN2* with a mutated methylation domain. **(G)** CLIP assays confirmed the interaction strength between *NSUN2* and the indicated mRNA in PANC-1 cells expressing either *NSUN2*-WT or *NSUN2* with a mutated methylation domain. **(H)** Immunoblot analysis of *NSUN2*, *CDCP1* and *STC1* protein expression levels in PANC-1 cells under the indicated treatments. **(I)** Total RNA isolated from treated PANC-1 cells was used for qRT-PCR analysis to assess the *CDCP1* and *STC1* mRNA expression. **(J-K)** Actinomycin D assay to assess the half-life of *CDCP1* **(J)** and *STC1* **(K)** mRNA in tumor cells. **(L)** PANC-1 Transwell migration/invasion: image panels (left) and measurements (right). Scale bar, 200 μm. **(M)** Neurite outgrowth was quantified under the indicated conditions in the Transwell co-culture model. Phase-contrast images were obtained at 6 h intervals. **(N)** (left) Representative fields from DRG co-cultures with tumor cells. (right) Summary statistics for tumor neurite invasion toward DRG. Scale bar, 500 μm. Each experiment was performed independently in triplicate, and all quantitative results are presented as mean ± SD. Statistical tests used for each panel were as follows: **(D, E, H)** one-way ANOVA; **(C)** two-way ANOVA; **(L, N)** unpaired t test; **(M)** paired t test. * for $P \leq 0.05$, ** for $P \leq 0.01$, *** for $P \leq 0.001$ and **** for $P \leq 0.0001$.

Figure S7. *NSUN2* K692 lactylation stabilizes *CDCP1*/*STC1* and promotes PNI.

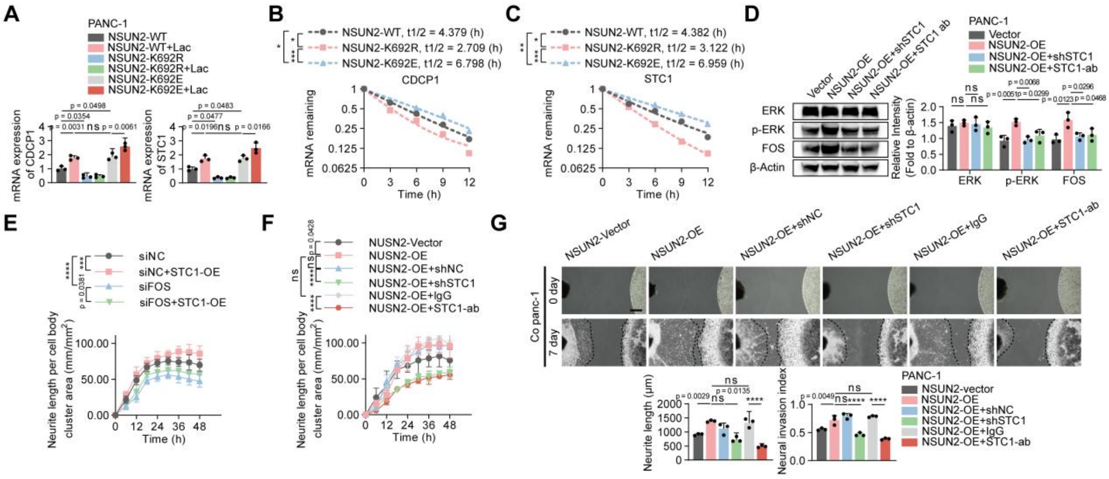

Figure S7. ***NSUN2*** **K692 lactylation stabilizes** ***CDCP1/STC1*** **and promotes PNI.**

(A) PANC-1 cells harboring the *NSUN2*-K692R or *NSUN2*-K692E mutant were generated using the CRISPR / Cas9 system, followed by treatment with or without L-lactate. Total RNA was extracted and subjected to qRT-PCR to assess the mRNA expression levels of *CDCP1* and *STC1*. **(B-C)** Actinomycin D assay to assess the half-life of *CDCP1* **(B)** and *STC1* **(C)** mRNA in PANC-1 cells. **(D)** Immunoblot analysis (left) and quantification analysis (right) of *ERK* / *FOS* signaling pathway in PANC-1 cells expressing vector or *NSUN2* overexpression plasmid, followed by treatment with or without transfection with *STC1* shRNA or *STC1* neutralizing antibody. **(E)** Neurite outgrowth was quantified under the indicated conditions in the Transwell co-culture model. The PANC-1 cells expressing siNC or *FOS* siRNA, followed by exposing to transfecting with *STC1* overexpression plasmid treatment. Phase-contrast images were obtained at 6 h intervals. **(F-G)** PANC-1 cells were expressing *NSUN2*-OE plasmid, followed by ± sh*STC1* and ± *STC1*-neutralizing antibody (isotype control), and subjected to subsequent experiments. **(F)** Neurite outgrowth was quantified under the indicated conditions in the Transwell co-culture model. Phase-contrast images were obtained at 6 h intervals. **(G)** (top) Representative fields from DRG co-cultures with tumor cells. (bottom) Summary statistics for tumor neurite invasion toward DRG. Scale bar, 500 μm. Each experiment was performed independently in triplicate, and all quantitative results are presented as mean ± SD. Statistical tests used for each panel were as follows: **(D, G)** one-way ANOVA; **(E, F)** two-way ANOVA. * for $P \leq 0.05$, ** for $P \leq 0.01$, *** for $P \leq 0.001$ and **** for $P \leq 0.0001$.

Figure S8. Quantification of immunofluorescence in vivo experiments related to PNI.

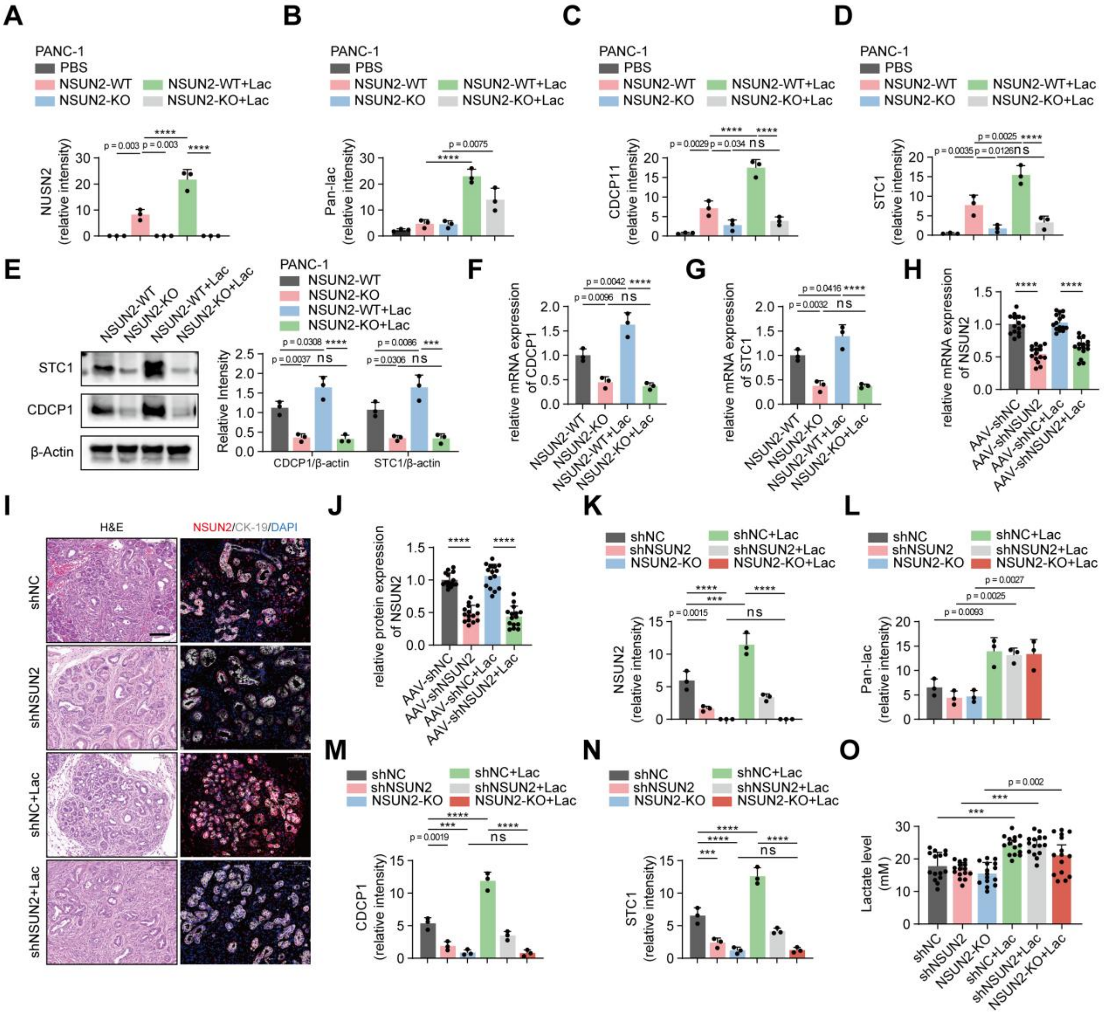

Figure S8. Quantification of immunofluorescence in vivo experiments related to PNI.

(A-D) Quantification of mIF images showing *NSUN2* **(A)**, Pan-lac **(B)**, *CDCP1* **(C)** and *STC1* **(D)** expression in sciatic nerve invasion model mice. **(E)** Immunoblot analysis (left) and quantification analysis (right) of *STC1* and *CDCP1* in tumor lysates from the sciatic-nerve invasion mouse model. **(F-G)** RT-qPCR analysis of *CDCP1* **(F)** and *STC1* **(G)** mRNA in tumor lysates from the sciatic-nerve invasion mouse model. **(H-O)** Following orthotopic AAV-mediated delivery of sh*NSUN2* in KPC mice with or without L-lactate treatment, tumor tissues were collected for subsequent experiments. **(H)** RT-qPCR quantification of *NSUN2* mRNA in tumors. **(I-J)** Immunofluorescence staining and quantification of *NSUN2* and *CK19* in tumors. **(K-N)** Quantification of mIF images showing *NSUN2* **(K)**, Pan-lac **(L)**, *CDCP1* **(M)** and *STC1* **(N)** expression in KPC model mice. **(O)** Lactate concentration in tumor tissues. Experiments shown in panels A-G and K-N were independently in triplicate (n = 3), whereas those in panels H-J and O involved fifteen biological replicates (n = 15), and all quantitative results are presented as mean ± SD. Statistical tests used for each panel were as follows: **(A-O)** one-way ANOVA. * for $P \leq 0.05$, ** for $P \leq 0.01$, *** for $P \leq 0.001$ and **** for $P \leq 0.0001$.

Table S1. Primers, probes and oligonucleotides used in the study.

Gene	Sequence(5'-3')	Application
NSUN2-F	CAAGCTGTTCGAGCACTACTAC	qRT-PCR
NSUN2-R	CTCCCTGAGAGCGTCCATGA	
CDCP1-F	CTGAACTGCGGGGTCTCTATC	qRT-PCR
CDCP1-R	GTCCCCAGCTTTATGAGAACTG	
STC1-F	GTGGCGGCTCAAAACTCAG	qRT-PCR
STC1-R	GTGGAGCACCTCCGAATGG	
GAPDH-F	ACAACTTTGGTATCGTGGAAGG	qRT-PCR
GAPDH-R	GCCATCACGCCACAGTTTC	
NSUN5-F	CGCTACCATGAGGTCCACTAC	qRT-PCR
NSUN5-R	GCATCTCGCACCACGTCTT	
NSUN6-F	TTAAGAGGAGCCCATGTCTATGC	qRT-PCR
NSUN6-R	CTTTGCGGCTTAGTTCAGAAATC	
NSUN7-F	GCATTGGCAAGATGTCGAATC	qRT-PCR
NSUN7-R	GGCCCTTAGTTCCTGTTTCCTA	
FOSL1-F	CAGGCGGAGACTGACAAACTG	qRT-PCR
FOSL1-R	TCCTTCCGGGATTTTGCAGAT	
Serpine1-F	ACCGCAACGTGGTTTTCTCA	qRT-PCR
Serpine1-R	TTGAATCCCATAGCTGCTTGAAT	
si/sh*NSUN2*-1-sense	CGAAUGAUGUGGACAACAA	siRNA/shRNA
si/sh*NSUN2*-1-antisense	UUGUUGUCCACAUCAUUCG	
si/sh*NSUN2*-2-sense	CAGUUUCUAGUUAGUGAAA	siRNA/shRNA
si/sh*NSUN2*-2-antisense	UUUCACUAACUAGAAACUG	
si*STC1*-sense	GGAUGUAUGACAUCUGUAAtt	siRNA
si*STC1*-antisense	UUACAGAUGUCAUACAUCCtt	
si*CDCP1*-sense	CCACGAGAAAGCAACAUUAtt	siRNA
si*CDCP1*-antisense	UAAUGUUGCUUUCUCGUGGtt	
si*LDHA*-1-sense	GCUGAUUUAUAAUCUUCUAtt	siRNA
si*LDHA*-1-antisense	UAGAAGAUUAUAAAUCAGCtt	
si*LDHA*-2-sense	GAAGAAGAGUGCAGAUACAtt	siRNA
si*LDHA*-2-antisense	UGUAUCUGCACUCUUCUUCtt	
si*LDHB*-1-sense	GGUUGCUCAGCUCAAGAAAtt	siRNA
si*LDHB*-1-antisense	UUUCUUGAGCUGAGCAACCtt	
si*LDHB*-2-sense	GGUUGAAAGUGCCUAUGAAtt	siRNA
si*LDHB*-2-antisense	UUCAUAGGCACUUUCAACCtt	
sgRNA-S1 (K692)	CGCCATCCGCATAAGACGAT	CRISPR-Cas9 genome editing (sgRNA)
sgRNA-S2 (K692)	CCGCCATCCGCATAAGACGA	CRISPR-Cas9 genome editing (sgRNA)
sgRNA-S3 (K692)	CCATCGTCTTATGCGGATGG	CRISPR-Cas9 genome editing (sgRNA)
ssODN-E (K692E)	ACGCTCTGCAGTGTCCCATCGTCTTATGC GGATGGCGGGGAAAGGCCTCCATTCGAACTT TTGTGCCCGAGAATGAACGGCTTCATTATCTC AGGATGATGGGGCTGGAGGTATTGGGAGAAA AGAAGAAGGAAG	HDR repair template (ssODN donor)

ssODN-R (K692R)	ACGCTCTGCAGTGTCCCATCGTCTTATGC GGATGGCGGGGAAAGGCCTCCATTCGAACTT TTGTGCCCAGAAATGAACGGCTTCATTATCTC AGGATGATGGGGCTGGAGGTATTGGGAGAAA AGAAGAAGGAAG	HDR repair template (ssODN donor)
ssODN-Q (K692Q)	ACGCTCTGCAGTGTCCCATCGTCTTATGC GGATGGCGGGGAAAGGCCTCCATTCGAACTT TTGTGCCCCAGAATGAACGGCTTCATTATCTC AGGATGATGGGGCTGGAGGTATTGGGAGAAA AGAAGAAGGAAG	HDR repair template (ssODN donor)
ssODN-A (K692A)	ACGCTCTGCAGTGTCCCATCGTCTTATGC GGATGGCGGGGAAAGGCCTCCATTCGAACTT TTGTGCCCGCGAATGAACGGCTTCATTATCTC AGGATGATGGGGCTGGAGGTATTGGGAGAAA AGAAGAAGGAAG	HDR repair template (ssODN donor)
NSUN2-geno-F	AAGGACCTGGCAAAGGGAAG	PCR genotyping/ Sanger validation
NSUN2-geno-R	ACGTCATTGTCTGGCTGTCC	
sgRNA (Exon 7)	TCTTGGCTTGATGGACGAGC	CRISPR-Cas9 RNP
ssODN (Exon 7)	AGGGATTTGTTATTGCGAATGATGTGGAC AACAAGCGCTGCTATCCTGCTCGTCCATCAAG CCAAGAGGCTGAGCAGCCCCTGCATCATGGT GGTCAACCATGATGCCTCCAGCATACCCAGGC TCCAGATAGA	HDR donor
NSUN2-geno-F	CCTGGCTCAAAGACCACACA	PCR genotyping/ Sanger validation
NSUN2-geno-R	CTCTTTCCTGCCGTCCACAT	
No m5C RNA (control)	AUGCAGUACGUAGCUGAUACGUCAG UCGACGUAGCUAGUCGAUCGUACGAUGC UAGUCGAUCGACGUCAGUCGAUGCAGUA CCGUAUGCUAGCUGAUCGUAGCUGAUCG ACGUAGCUGACGUAGCUAGCUGAUCGAC GUAGCUGAUCGACGUCGAUCGACG UAG	Negative control RNA (dot blot specificity)

Table S2. Clinical and pathologic variables

Clinical variables		
Age, median (range), year		59.73 (37-79)
Gender, male/female, male%		86/56, 60.56%
Preoperative CEA, median (range), ng/mL		7.06 (0.20-314.50)
Preoperative CA19–9, median (range), U/mL		450.82 (0.60-6816.00)
Survival Status, survival/dead, survival%		21/121, 14.79%
Pathologic variables		
Histology differentiations, caes (%)		
	Well	11 (7.75)
	Moderate	65 (45.77)
	Poor	66 (46.48)
T stage, cases (%)		
	T1	9 (6.34)
	T2	12 (8.45)
	T3	121 (85.21)
TNM stage, cases (%)		
	IA	6 (4.23)
	IB	7 (4.93)
	IIA	48 (33.80)
	IIB	81 (57.04)
Lymph_Node (+), cases (%)		81 (57.04)
Perineural_Invasion (+), cases (%)		116 (81.69)

Table S3. Antibody and Kit

Company	Antibody	Art.No.	Species	Application and Dilution		
Immunofluorescence Antibody						
Jingjie	Anti-L-Lactyl Lysine Rabbit mAb	PTM-1401RM	Rabbit	WB 1:500-1:1000	IHC-P 1:50-1:100	ICC/IF 1:50-1:100
CST	*β3-Tubulin*	D65A4	Rabbit	WB 1:1000	IHC-P 1:50	IP 1:50
CST	Keratin 17/19	D4G2	Rabbit	WB 1:1000	IHC-P 1:1200	IF 1:50
abcam	*STUB1*	ab134064	Rabbit	WB 1:10000-1:50000	IHC-P 1:100	IF/ICC 1:250-1:500
proteintech	*NSUN2*	20854-1-AP	Rabbit	WB 1:5000-1:50000	IHC-P 1:50-1:500	IF/ICC 1:750-1:3000
proteintech	*CDCP1*	12754-1-AP	Rabbit	WB 1:500-1:2000	IHC-P 1:50-1:500	
proteintech	*STC1*	20621-1-AP	Rabbit	WB 1:500-1:1000	IHC-P 1:50-1:500	
Western blotting Antibody						
proteintech	*B-Actin*	20536-1-AP	Rabbit	WB 1:4000-1:10000	IHC-P 1:50-1:500	IF/ICC 1:200-1:800
proteintech	*NSUN5*	15449-1-AP	Rabbit	WB 1:500-1:3000	IHC-P 1:50-1:500	
proteintech	*NSUN6*	17240-1-AP	Rabbit	WB 1:500-1:2000	IHC-P 1:500-1:2000	
proteintech	ubiquitin	10201-2-AP	Rabbit	WB 1:1000-1:8000	IHC-P 1:50-1:500	IF/ICC 1:200-1:800
proteintech	5-methylcytosine	68301-1-Ig	Mouse	Dot Blot 1:2500-1:10000	IHC-P 1:2500-1:10000	
proteintech	ERK1/2	11257-1-AP	Rabbit	WB 1:2000-1:16000		
proteintech	Phospho-ERK1/2	80031-1-RR	Rabbit	WB 1:2000-1:10000		
proteintech	c-Fos	66590-1-Ig	Mouse	WB 1:5000-1:50000		
UpingBio	*NSUN7*	YP-Ab-11882	Rabbit	WB 1:500-1:2000		
Immunoprecipitation Antibody						
abcam	*NSUN2*	ab259941	Rabbit	IP 1:30	IHC-P 1:200	ICC/IF 1:1000
abcam	5-methylcytosine	ab10805	Mouse	IP 1:50		
CST	DYKDDDDK Tag	D6W5B	Rabbit	WB 1:1000	IHC-P 1:400-1:1600	IP 1:50
CST	HA-Tag	C29F4	Rabbit	WB 1:1000	IHC-P 1:800-1:3200	IP 1:50
Kit						
Vazyme, China	PrimeScript RT Reagent Kit					
Panovue, China	PANO 4-plex IHC Kit					
EZB Bioscience, USA	Universal RNA Purification Kit					
Millipore, USA	EZ-Magna RIP kit					
Ambion, USA	Dynabeads mRNA Purification Kit					
Solarbio, China	Lactate Assay Kit					

Table S4 Univariate and multivariate analysis of Overall Survival (OS) in PDAC patients (n = 142)

Variables	Characteristics	Univariate analysis			Multivariate analysis		
		HR	95% CI	p value	HR	95% CI	p value
**Age**	< 60 (ref)						
	≥ 60	1.013	0.846-1.214	0.886			
**Gender**	Female (ref)						
	Male	1.027	0.854-1.235	0.779			
**Differentiation**	Well (ref)						
	Moderate	1.219	0.856-1.736	0.273	0.983	0.687-1.406	0.923
	Poor	2.184	1.532-3.112	<0.001***	1.776	1.222-2.58	0.003**
**TNM stage**	I (ref)						
	II	3.899	1.933-7.864	<0.001***	3.061	1.479-6.336	0.003**
**T stage**	I (ref)						
	II	1.246	0.759-2.045	0.385			
	III	1.464	1.005-2.133	0.047*			
**Lymph node metastasis**	Negative (ref)						
	Positive	2.012	1.375-2.946	<0.001***			
**Perineural invasion**	Negative (ref)						
	Positive	2.195	1.602-3.008	<0.001***	1.421	1.012-1.994	0.043*

Abbreviations: HR = hazard ratio; 95% CI = 95% confidence interval; TNM = tumor node metastasis; ref = reference. As lymph node metastasis and T stage are included in the TNM stage, we didn't include it in the multivariate analysis. Cox regression analysis, * $p < 0.05$, ** $p < 0.01$, *** $p<0.001$.

Table S5 Univariate and multivariate analysis of Disease-free Survival (DFS) in PDAC patients (n = 142)

Variables	Characteristics	Univariate analysis			Multivariate analysis		
		HR	95% CI	p value	HR	95% CI	p value
**Age**	< 60 (ref)						
	≥ 60	1.013	0.847-1.213	0.885			
**Gender**	Female (ref)						
	Male	1.042	0.868-1.252	0.657			
**Differentiation**	Well (ref)						
	Moderate	1.202	0.845-1.71	0.307	1.015	0.704-1.463	0.936
	Poor	2.129	1.496-3.03	<0.001***	1.753	1.197-2.566	0.004**
**TNM stage**	I (ref)						
	II	4.511	2.206-9.226	<0.001***	4.013	1.833-8.784	<0.001***
**T stage**	I (ref)						
	II	1.222	0.744-2.005	0.429			
	III	1.515	1.04-2.206	0.03*			
**Lymph node metastasis**	Negative (ref)						
	Positive	2.152	1.467-3.158	<0.001***			
**Perineural invasion**	Negative (ref)						
	Positive	2.275	1.66-3.118	<0.001***	1.484	1.056-2.085	0.023*

Abbreviations: HR = hazard ratio; 95% CI = 95% confidence interval; TNM = tumor node metastasis; ref = reference. As lymph node metastasis and T stage are included in the TNM stage, we didn't include it in the multivariate analysis. Cox regression analysis, * $p < 0.05$, ** $p < 0.01$, *** $p<0.001$.
